# Potential mechanisms of osthole against bladder cancer cells based on network pharmacology, molecular docking, and experimental validation

**DOI:** 10.1186/s12906-023-03938-5

**Published:** 2023-04-17

**Authors:** Yunzhong Jiang, Mengzhao Zhang, Lu Wang, Lu Zhang, Minghai Ma, Minxuan Jing, Jianpeng Li, Rundong Song, Yuanquan Zhang, Zezhong Yang, Yaodong Zhang, Yuanchun Pu, Xiaowei Qu, Jinhai Fan

**Affiliations:** 1 Department of Urology, the First Affiliated Hospital of Xi’an Jiaotong University, Xi’an, China; 2 Department of Vascular Surgery, the First Affiliated Hospital of Xi’an Jiaotong University, Xi’an, China; 3 Department of Geriatrics, the Yan’an University Xianyang Hospital, Xian’yang, China; 4 Oncology Research Lab, Key Laboratory of Environment and Genes Related to Diseases, Ministry of Education, Xi’an, China

**Keywords:** Osthole, Bladder cancer, Network Pharmacology, Molecular docking

## Abstract

**Background:**

Osthole was traditionally used in treatment for various diseases. However, few studies had demonstrated that osthole could suppress bladder cancer cells and its mechanism was unclear. Therefore, we performed a research to explore the potential mechanism for osthole against bladder cancer.

**Methods:**

Internet web servers SwissTargetPrediction, PharmMapper, SuperPRED, and TargetNet were used to predict the Osthole targets. GeneCards and the OMIM database were used to indicate bladder cancer targets. The intersection of two target gene fragments was used to obtain the key target genes. Protein–protein interaction (PPI) analysis was performed using the Search Tool for the Retrieval of Interacting Genes (STRING) database. Furthermore, we used gene ontology (GO) and Kyoto Encyclopedia of Genes and Genomes (KEGG) pathway enrichment analyses to explore the molecular function of target genes. AutoDock software was then used to perform molecular docking of target genes,osthole and co-crystal ligand. Finally, an in vitro experiment was conducted to validate bladder cancer inhibition by osthole.

**Results:**

Our analysis identified 369 intersection genes for osthole, the top ten target genes included *MAPK1, AKT1, SRC, HRAS, HASP90AA1, PIK3R1, PTPN11, MAPK14, CREBBP*, and *RXRA*. The GO and KEGG pathway enrichment results revealed that the PI3K-AKT pathway was closely correlated with osthole against bladder cancer. The osthole had cytotoxic effect on bladder cancer cells according to the cytotoxic assay. Additionally, osthole blocked the bladder cancer epithelial-mesenchymal transition and promoted bladder cancer cell apoptosis by inhibiting the PI3K-AKT and Janus kinase/signal transducer and activator of transcription (JAK/STAT3) pathways.

**Conclusions:**

We found that osthole had cytotoxic effect on bladder cancer cells and inhibited invasion, migration, and epithelial-mesenchymal transition by inhibiting PI3K-AKT and JAK/STAT3 pathways in in vitro experiment. Above all, osthole might have potential significance in treatment of bladder cancer.

**Subjects:**

Bioinformatics, Computational Biology, Molecular Biology.

**Supplementary Information:**

The online version contains supplementary material available at 10.1186/s12906-023-03938-5.

## Introduction

Bladder cancer is the seventh most common cancer in men, wherein urothelial carcinoma is the most prevalent type of cancer that is histologically diagnosed. Statistically, the age-standardized incidence rate per 100,000 people per year is 9.5 for men and 2.4 for women, worldwide [1]. Surgery and chemotherapy are the primary treatments for bladder cancer. However, the current chemotherapy drug protocols are centered on gemcitabine and cisplatin, both of which are costly and have many negative side effects for patients [2, 3]. There are, however, established traditional Chinese medicines with notably high efficacy and low toxicity that are widely used in the treatment of various diseases [4–6]. Osthole, a compound extracted from the fruit of *Fructus cnidii* and other plants had a long history in China. It had postive effect in invigorating kidney and strengthening yang, which was recorded in Compendium of Materia Medica. Osthole was traditionally used in treatment for man impotence,blood stasis, ringwom, and foul disease (Editorial Committee of Chinese Pharmacopoeia, 2010). In modern world, osthole was widely used in nerve protection, anti-inflammation, and anti-oxidation, anti-osteoporosis and anti-allergic, which was proved by in vivo and in vitro expreiments [7, 8]. Recenlty, it was reported that osthole could exhibit substantial inhibitory effects in various cancers, including lung cancer, prostate cancer, and ovarian cancer [9–11]. However, identification of the targets for osthole was the key step in the treatment of various cancers. Network pharmacology was a effective tool to explore the potential targets of drugs. It emphasized the regulation of multi-pathway and network between diseases and drugs, which could improve the therapeutic effect of drugs and reduce the toxic side effects [12]. Only a few studies have reported on the inhibitory effects of osthole in bladder cancer and the mechanism of osthole effects against bladder cancer is poorly understood. Therefore, we aimed to demonstrate that osthole inhibited bladder cancer cells and to reveal the underlying mechanism of the activities of osthole in bladder cancer treatment by using the method of Network pharmacology.

## Materials and methods

### Prediction of target genes for osthole and bladder cancer

The three-dimensional (3D) chemical structure of osthole was downloaded from PubChem [13] (https://pubchem.ncbi.nlm.nih.gov/). In order to predict osthole targets, the SwissTargetPrediction [14] (http://www.swisstargetprediction.ch/), PharmMapper [15] (http://www.lilab-ecust.cn/pharmmapper/), TargetNet [16] (http://targetnet.scbdd.com/calcnet/index/), and Super-PRED [17] (https://prediction.charite.de/) databases were consulted. The targets of bladder cancer were also obtained from GeneCards [18] (https://www.genecards.org/) and OMIM [19] (https://www.omim.org/). Additionally, the Venny 2.1 online tool (https://bioinfogp.cnb.csic.es/tools/venny/index.html) was used to obtain target gene intersections.

#### Construction of PPI network

The STRING data base [20] (https://cn.string-db.org/) was used to construct the protein–protein interaction (PPI) network in select target genes. The isolated target genes with the highest confidence level (0.95) were extracted. The selected target genes were then imported using Cytoscape 3.9.0 software to demonstrate the gene interactions.CytoHubba was used to calculate the degree of other target genes. Finally, the top ten target genes were screened and selected based on the PPI network results.

#### Gene function and pathway enrichment analysis

After obtaining the intersection of target genes. The “ClusterProfiler” R package was installed in R (v.4.1.2) software in order to perform gene ontology (GO) and Kyoto Encyclopedia of Genes and Genomes (KEGG) pathway enrichment analyses for target genes [21, 22]. Then, we selected the top 10 terms associated with BP (Biological Process),CC (Celluar Component) and MF (Molecular Function) according to the *p* value (*p* < 0.05) and gene count. Furthermore, the top of 10 pathways was also selected according the results of KEGG enrichment analyses. At last, the “ggplot2” R package was used to visualize the top ten terms of the results of enrichment analyses [23].

#### Molecular docking analysis

The protein data bank (PDB) files for the target proteins was downloaded from on the Research Collaboratory for Structural Bioinformatics website (https://www.rcsb.org/) [24]. The structure of co-crystal ligands were downloaded from Pubchem (https://pubchem.ncbi.nlm.nih.gov/) [13]. Then, the molecular visualization system PyMOL2 (https://pymol.org/2/) was used to remove water and organic ligand from the target proteins and the result was saved in the PDBQT file format [25]. AutoDock 1.5.7 software was then used to add all the polar hydrogens and target protein was chosen as a receptor [26]. The 3D structure of osthole was downloaded from PubChem (https://pubchem.ncbi.nlm.nih.gov/). Additional optimizations included adding hydrogen, detecting and choosing torsion linkage and regarding osthole as ligand, using AutoDock 1.5.7 software. The grid box parameter was set and the semi-flexible docking of osthole and target proteins was performed. In addition, we also validated our docking protocol by re-docking the co-crystalized ligands into their corresponding pockets. Finally, PyMOL 2 was used to visualize the docking results. We used different color to mark the protein (blue) and ligand (red). Furthermore, the rod like structure of amino acid interacted with osthole was marked as orange. “pheatmap” R package was used to show the binding energy between ligands and target proteins [27].

#### Experimental in vitro validation

##### Cell lines

Human bladder cancer (5637 and 253 J), and normal human urothelial (SV-HUC-1) cell lines were purchased from American Type Culture Collection (ATCC, Rockville, Maryland, USA). The 5637 cell lines were cultured in a RPMI-1640 medium supplemented with 10% fetal bovine serum (FBS; Biological Industries, USA). The 253 J cell line was cultured in Dulbecco’s modified eagle’s medium (DMEM) supplemented with 10% FBS (Biological Industries, USA). The SV-HUC-1 cell line was cultured in Ham's F-12 K medium supplemented with 10% FBS (Biological Industries, USA). All cell lines were cultured at 37 ℃, aired with 5% CO2 and 95% humidity in a cell incubator.

#### Chemicals and reagents

Tetrazolium compound (3-[4,5-dimethyl-2-thiazolyl]-2,5-diphenyl-2H-tetrazolium bromide; MTT) was purchased from Sigma-Aldrich (St. Louis, MO, USA). Osthole (Solarbio,

SO8130) was dissolved in dimethyl sulfoxide (DMSO; Sigma, St. Louis, MO, USA) in order to obtain specific concentrations of osthole for experiments. Cisplatin (NSC119875) was obtained from Selleck (https://www.selleck.cn/) and dissolved in deionized water. All media and FBS were purchased from Thermo Fisher Scientific (Waltham, MA, USA). Transwell chambers with an 8-μm pore size were purchased from Millipore (Darmstadt, Germany).

#### Cytotoxic assay

The cytotoxic effect of osthole was determined by MTT assay. Cell lines 5637, 253 J, and SV-HUC-1 were seeded in 96-well plates at 5 × 10^3^ cells with 200 μL medium per well. Once the cell density reached 70%, it was exposed to different concentrations of Osthole (25–200 μM) for 24 h and 48 h respectively. Furthermore, we also used cisplatin (2.5-20 μM) as the positive control. Then, MTT (5 mg/mL) was added to 200 μL of the medium per well for a further 4 h. The MTT solution was then removed, prior to adding 150 μL dimethyl sulfoxide per well and the plates were shaken for 10 min. Finally, optical density value was detected at 490 nm using a plate reader (Bio-Rad, Hercules, CA, USA). The experiment was performed in triplicate [5].

#### Cell colony formation assay

The 5637 and 253 J cells were seeded in 6-well plates at 1 × 10^3^ cells per well. After 48 h of incubation, we treated cancer cells with different concentrations (0, 50, and 100 μM). After 10 days, chilled phosphate-buffered saline (PBS) was used to wash every well carefully. Then, 4% paraformaldehyde and 1 × crystal violet was used to fix and dye the cell colonies. Finally, PBS was used to wash the wells until the cell colony could be observed with naked eye. The experiment was performed in triplicate [28].

#### Cell flow cytometry assays

Cancer cell apoptosis and cell cycle arrest were analyzed using the cell flow cytometry assays. To detect cell apoptosis, 5637 and 253 J cells were seeded into 6-well plates at 30 × 10^4^ cells per well for 24 h. The cells were then treated at different concentrations (0, 50, and 100 μM) for 24 h. Chilled PBS was used to wash the 6-well plates, prior to suspension with binding buffer. The Annexin V-FITC apoptosis detection kit (BD Biosciences, San Jose, CA, USA) and FACSCalibur flow cytometry (BD Biosciences, San Jose, CA, USA) were used to detect and calculate the apoptosis rate of the cancer cells. For detecting cell cycle arrest, 5637 and 253 J cells were seeded into 6-well plates and treated at two different concentrations (0, 50, and 100 μM) for 24 h. Cells were washed with chilled PBS and collected using a centrifugal method and subsequently fixed with 70% ethanol at 4 °C overnight. Cell samples were then incubated using propidium iodide (PI) and RNase. FACSCalibur flow cytometry (BD Biosciences, San Jose, CA, USA) was then used to identify cell cycle arrest and calculate the number of cells present in the different cell cycle stages. The experiment was performed in triplicate [28].

#### Wound healing assay

The 5637 cells and 253 J cells were seeded into 6-well plates and a 200-μL-pipette tip was used to make scratches prior to PBS washing of every well. Osthole solution was diluted in serum-free medium to added to each well when the confluency approached 100%. The cells were treated at different concentration (0, 25,and 50 μM). An inverted microscope at 200X magnification was used to observe the state of scratch healing and take photographs. The experiment was performed in triplicate [29].

#### Transwell migration assays

The 5637 cells and 253 J cells were seeded into 6-well plates and left for 24 h. Then, every 6-well plate was treated with two different concentrations (25 and 50 μM) for 24 h. Cells from each well were collected in 1.5 mL tubes and suspended in a medium contained 0.4% FBS. Concurrently, 5 × 10^4^ of 5637 and 253 J cells were added into 200 μL medium contained 0.4% FBS and seeded into the transwell upper chamber. In the transwell lower chamber, 800 μL medium with 10% FBS was added to the cells. After 24 h incubation, the lower chamber was washed with PBS and fixed with 4% paraformaldehyde. The cells were then visualized in the lower chambers using 1 × crystal violet. Finally, an inverted light microscope at 200X magnification was used to capture photographs. The experiment was performed in triplicate [29].

#### Western blot

After being treated with three different concentrations (25, 50, and 100 μM), 5637 and 253 J cells were washed with PBS three times. After cell washing, radioimmunoprecipitation assay buffer (Beyotime Institute of Biotechnology, Jiangsu, China) with 0.1 M phenylmethanesulfonylfluoride was added into each well and the well plates placed on ice for 10 min. Cells were then scraped into 1.5 mL tubes and the samples were ultracentrifuged at 15,000 rpm for 15 min at 4 ℃ to obtain a protein supernatant. The BCA protein quantification assay (Thermo Fisher Scientific, USA) was used to quantify the protein samples. Western blot was performed using protein samples (20 μg). After 10% sodium dodecyl sulfate–polyacrylamide gel electrophoresis (SDS-PAGE) separation, protein molecules were transferred into polyvinylidene fluoride (PVDF) membranes for two hours. Then, 5% defatted milk was used to block PVDF membranes for 1 h at room temperature and the PVDF membranes were incubated with primary antibodies at 4 ℃ overnight. After 18–24 h, polysorbate 20 tris-buffered saline (TBST) was used to wash the PVDF membranes three times. The secondary antibodies were incubated with washed PVDF membranes for 1 h at room temperature. All antibodies used in western blot was shown in Table 1. Finally, the electrogenerated chemiluminescent (ECL) detection system.

**Table 1:** Primary antibodies used in western blot

antibody	species	company	dilution rate
Cleaved caspase-3	Rabbit monoclonal	CST 9661	1:1000
caspase-3	Rabbit monoclonal	CST 14,220	1:1000
Cleaved-PARP	Rabbit monoclonal	CST 5625	1:1000
Bcl-2	Mouse monoclonal	CST 17,447	1:1000
Bax	Rabbit monoclonal	CST 41,662	1:1000
β-actin	Mouse Monoclonal	Proteintech 60,008–1-Ig	1:1000
E-cadherin	Rabbit monoclonal	CST 3195	1:1000
N-cadherin	Rabbit monoclonal	CST 13,116	1:1000
P-stat3	Rabbit monoclonal	CST 9145	1:1000
Stat3	Rabbit monoclonal	CST 12,640	1:1000
AKT	Rabbit monoclonal	CST 4685	1:1000
P-akt	Rabbit monoclonal	CST 4060	1:1000
C-myc	Rabbit monoclonal	CST 18,583	1:1000
Snail	Rabbit monoclonal	CST 3879	1:1000
p-mtor	Rabbit monoclonal	CST 5536	1:1000
JAK1	Rabbit monoclonal	CST 29,261	1:1000
MMP2	Rabbit polyclonal	Proteintech 10,373–2-AP	1:1000
MMP9	Rabbit polyclonal	Proteintech 10,375–2-AP	1:1000
CyclinB1	Rabbit monoclonal	CST 12,231	1:1000
CDK2	Rabbit polyclonal	Proteintech 10,122–1-AP	1:1000
CDC2	Rabbit monoclonal	CST 28,439	1:1000
Secondary Antibodies	Goat Anti-Rabbit IgG	Proteintech SA00001-2	1:2000
Secondary Antibodies	Goat Anti-Mouse IgG	Proteintech SA00001-1	1:2000

(Bio- Rad Laboratories, CA, USA) and developing solution were used to visualize protein bands [29].

#### Statistical analysis

All experiments were performed at least three times, and the results of the experiments were shown as mean ± standard error of the mean (SEM). We used Student's t-test to compare difference between two dependent groups. *P* < 0.05 was considered statistically significantly.

## Results

### Target predictions for osthole and bladder cancer

**Fig. 1:**
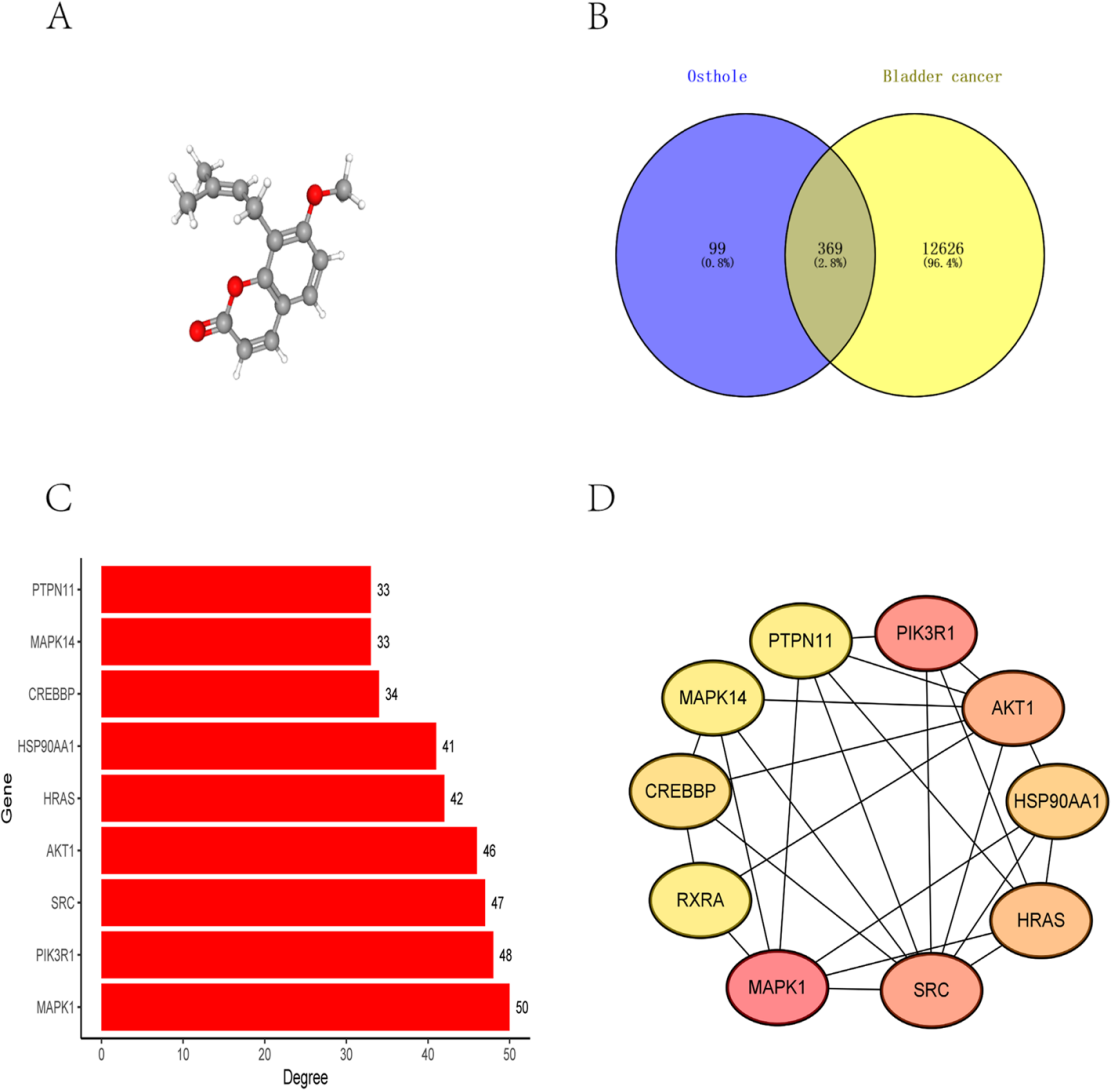
The prediction of targets for osthole against bladder cancer. **A** The 3D chemical structure of osthole **B** Venn diagram of potential targets of osthole against bladder cancer. **C** The barchart of the top ten target genes arranged by degree. **D** The PPI network of top ten target genes

The 3D chemical structure obtained for osthole is illustrated in Fig. 1A. Additionally, 468 target genes for osthole and 12,995 target genes for bladder cancer were obtained. Following intersection, 369 target genes were obtained as the final target genes (Fig. 1B).

#### Construction of PPI network.

The 369 target genes obtained were imported into the STRING database to perform protein interaction analysis; the 365 nodes and 1163 edges obtained are presented in Supplementary Figure 1. Then, the PPI network data was imported into the Cytoscape software. The top ten target genes were screened out based on the degree: *MAPK1, AKT1, SRC, HRAS, HASP90AA1, PIK3R1, PTPN11, MAPK14, CREBBP,* and *RXRA* (Fig. 1C). The top ten target genes network is displayed in Fig. 1D.

#### Results from GO and KEGG pathway analyses

**Fig. 2:**
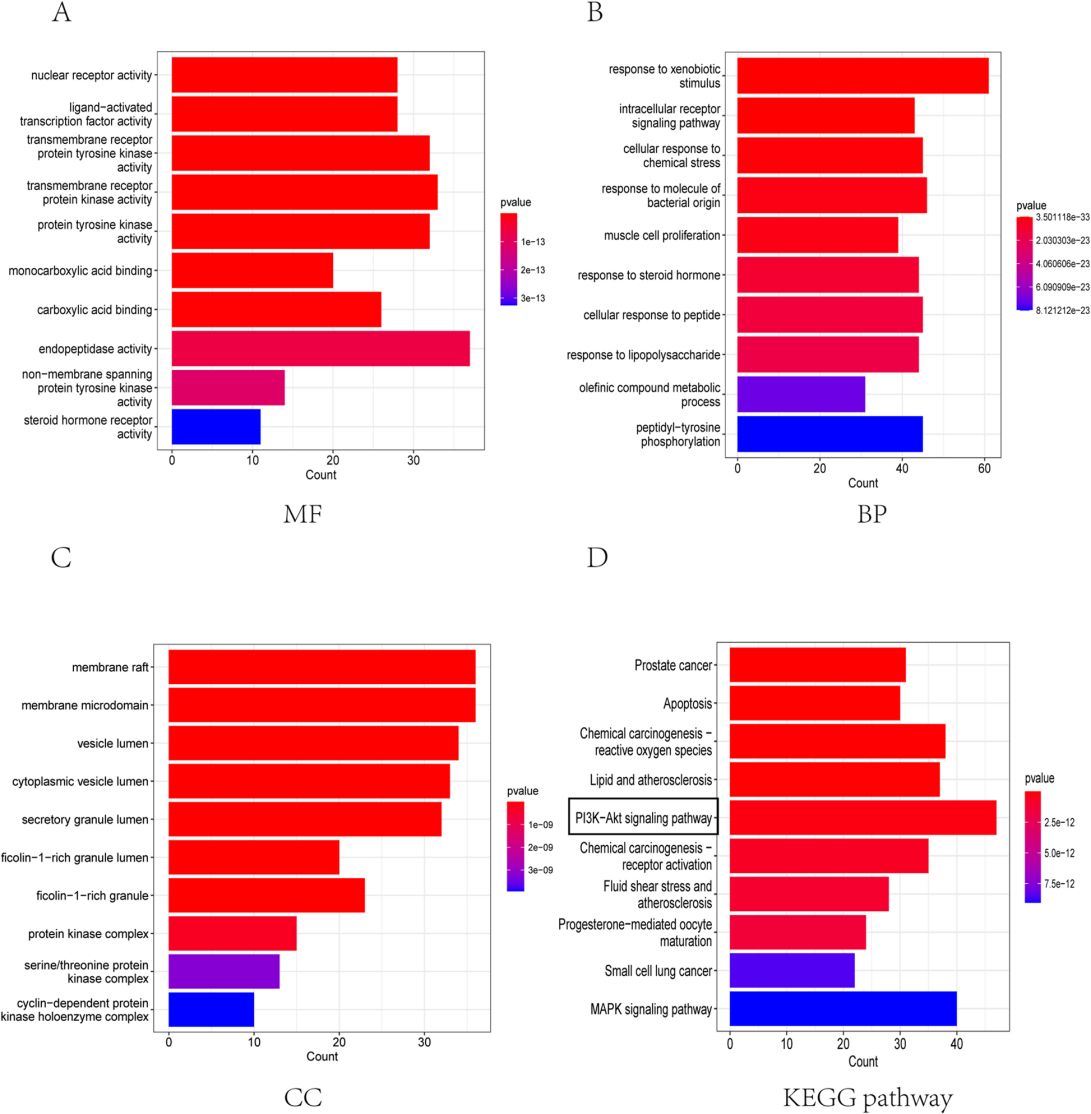
GO and KEGG pathway enrichment analysis. **A** The barchart of top ten molecular function in GO enrichment analysis. **B** The barchart of top ten biological process in GO enrichment analysis. **C** The barchart of top ten celluar component in GO enrichment analysis. **D** The barchart of top ten signal pathways in KEGG enrichment analysis

Using “ClusterProfiler” R package, GO and KEGG pathway analysis was performed. For GO enrichment analysis, the target genes were found to be closely correlated with endopeptidase activity, a transmembrane receptor protein that is actively involved in molecular function (Fig. 2A). Furthermore, the target genes were related in their response to xenobiotic stimulation, muscle cell proliferation, and cellular response to peptide in biological processes (Fig. 2B). In addition, the target genes revealed to have a strong relationship with cell membrane cellular components (Fig. 2C). From KEGG pathway enrichment analysis, the PI3K-AKT pathway was determined to be closely related to the target genes (Fig. 2D).

#### Molecular docking analysis

**Fig. 3:**
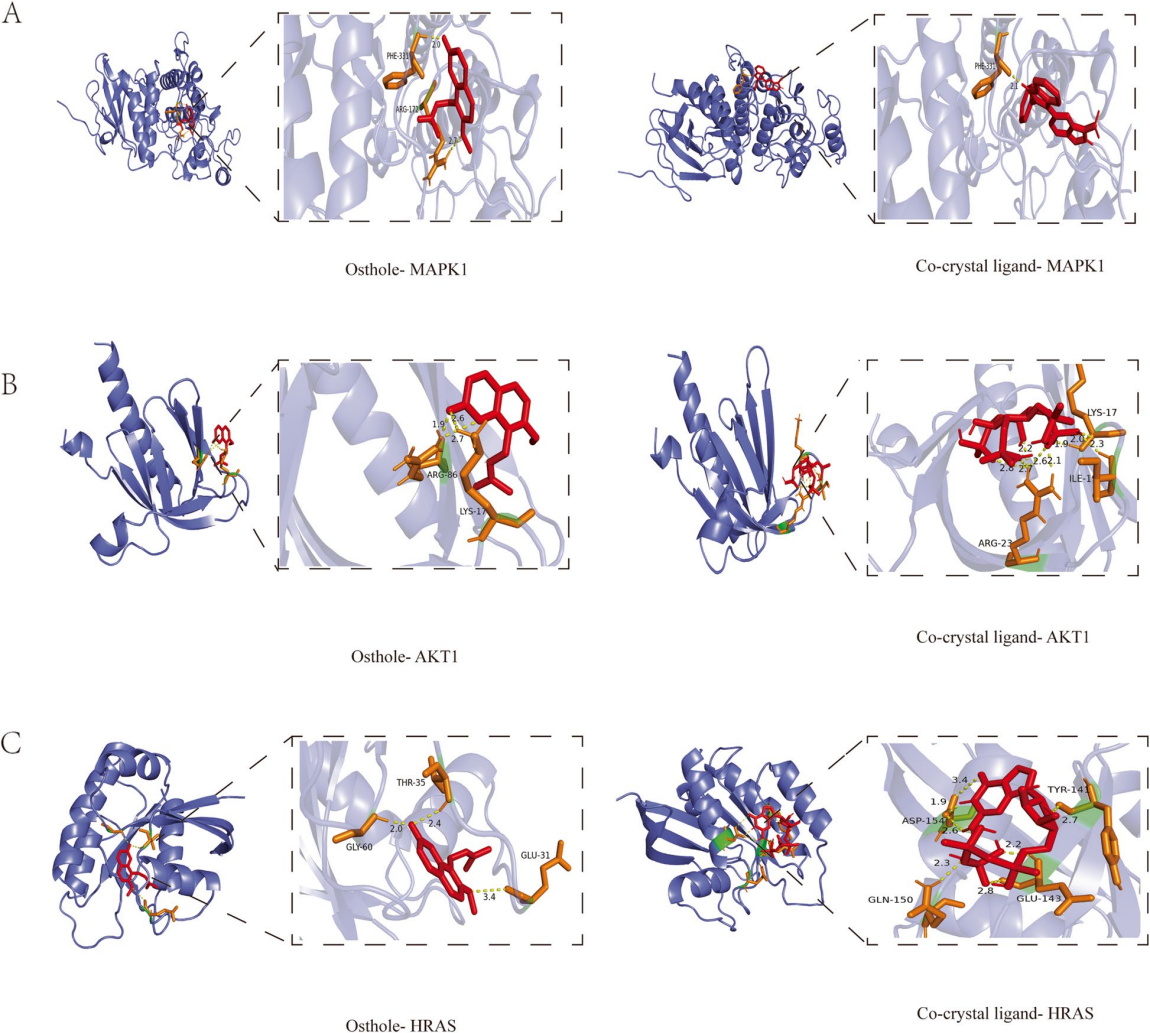
Molecular docking of ligands and target proteins. **A** Docking process of osthole, co-crystal ligand with MAPK1 protein. **B** Docking process of osthole, co-crystal ligand with AKT1 protein. **C** Docking process of co-crystal ligand with HRAS protein

**Fig. 4:**
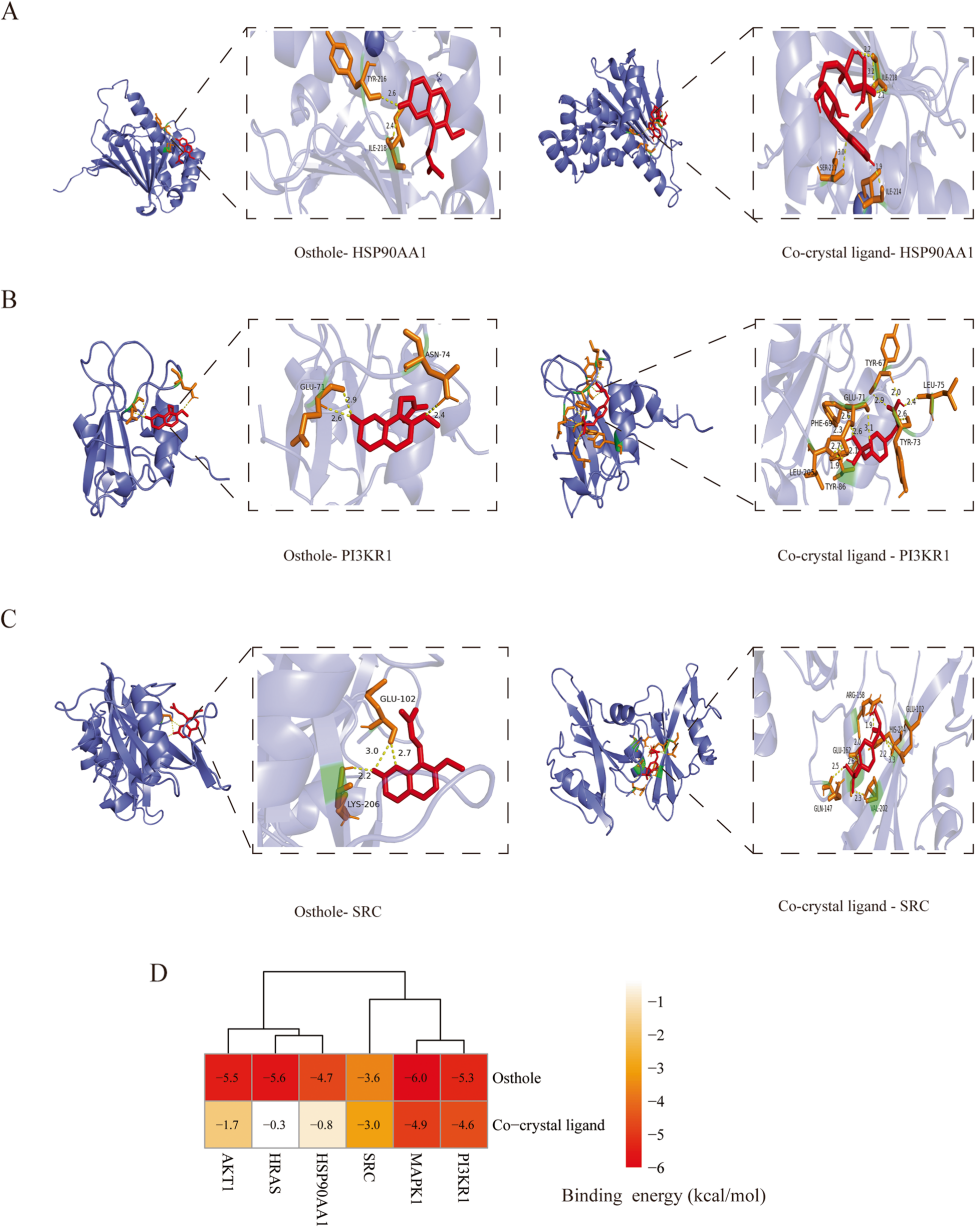
Molecular docking of ligands and target proteins. **A** Docking process of osthole, co-crystal ligand with HSP90AA1 protein. **B** Docking process of osthole, co-crystal ligand with PI3KR1 protein. **C** Docking process of osthole, co-crystal ligand with SRC protein. **D** Heatmap of binding energy between ligands and target proteins

According to the results of PPI network analysis of the top ten target genes, we selected the top six target genes to perform molecular docking, including *MAPK1, SRC, PIK3R1, HRAS, HSP90AA1*, and *AKT1*. From the results of molecular docking, the six target proteins were combined with osthole through the hydrogen bonds. Osthole was interacted with ARG172, PHE331 of MAPK1 protein on the A chain through two hydrogen bonds (Fig. 3A). Osthole was combined with ARG86, LYS17 of AKT1 protein on the A chain through three hydrogen bonds (Fig. 3B). Osthole formed three hydrogen bonds with GLY60, GLU31, THR35 on the A chain of HRAS protein (Fig. 3C). Osthole formed an interaction with TYR216, ILE218 of HSP90AA1 protein on the A chain through two hydrogen bonds (Fig. 4A). Osthole was interacted with GLU71, ASN74 on the A chain of PI3KR1 protein through three hydrogen bonds (Fig. 4B). Osthole was combined with SRC protein through three hydrogen bonds interacted with GLU102, LYS206 on the A chain of target protein (Fig. 4C). In addition, we also performed molecular docking between target proteins and its co-crystal ligands in order to validate our docking protocol (Figs. 3, 4). The structure of co-crystal ligands were shown in Supplementary Figure 2. The PDB identification, AutoDock-calculated binding energy between ligands and proteins and the size of docking box were shown in Table 2. It was obvious that osthole had a good binding affinity to target proteins compared with its co-crystal ligands (Fig. 4D). The binding energy of osthole was lower than the co-crystal ligands, which validated our docking protocols.

**Table 2:** Molecular docking of Osthole and co-crystal ligands to targets proteins

Compounds	Targets	Binding energy (kcal/mol)	PDB ID	Box size (x,y,z)
Osthole	MAPK1	-6.0	1TVO	(66,44,66)
Co-crystol ligand	MAPK1	-4.9	1TVO	(66,44,66)
Osthole	AKT1	-5.5	2UZS	(48,40,40)
Co-crystol ligand	AKT1	-1.7	2UZS	(48,40,40)
Osthole	HRAS	-5.6	121P	(40,40,44)
Co-crystol ligand	HRAS	-0.3	121P	(40,40,44)
Osthole	HSP90AA1	-4.7	1BYQ	(48,54,74)
Co-crystol ligand	HSP90AA1	-0.8	1BYQ	(48,54,74)
Osthole	PI3KR1	-5.3	1PIC	(48,40,40)
Co-crystol ligand	PI3KR1	-4.6	1PIC	(48,40,40)
Osthole	SRC	-3.6	1A07	(40,40,66)
Co-crystol ligand	SRC	-3.0	1A07	(40,40,66)

### Osthole had cytotoxic effect on bladder cancer cells and inhibits colony formation in vitro

**Fig. 5:**
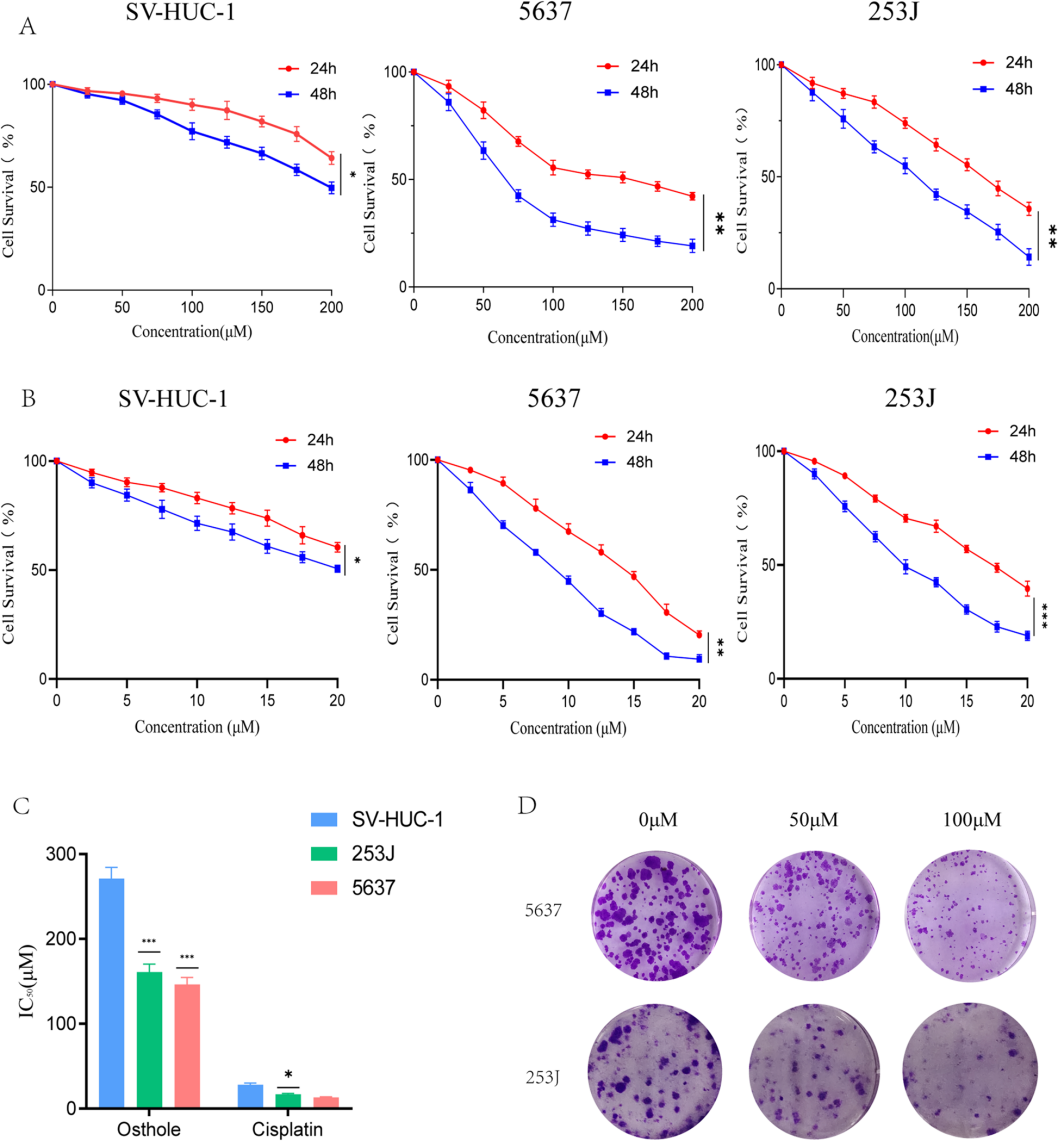
Osthole inhibits the colony formation, and induce apoptosis of bladder cancer cell in vitro. **A** SV-HUC-1, 253 J and 5673 cell lines were treated with osthole and its cell survival was estimated by MTT assays. **B** Cisplatin, as the positive control was added to three cell lines. SV-HUC-1, 253 J and 5673 cell survival was estimated by MTT assays. **C** The IC_50_ of three cell lines treated with osthole or cisplatin for 24 h. **D** The images of colony formation assays. *p < 0.05, **p < 0.01, ***p < 0.001 compared to the control group (0 μM)

According to the cytotoxic assay, osthole had cytotoxic effect on bladder cancer cells in dose- and time-dependent manner (Fig. 5A). Furthermore, cisplatin could also had cytotoxic effect on bladder cancer cell in dose-and time-dependent manner as the positive control (Fig. 5B). The IC_50_ of different cell line treated with osthole or cisplatin were shown in Table 3. We discovered that the IC_50_ of SV-HUC-1 treated with osthole for 24 h and 48 h were 271.1 ± 13.1 μM and 211.2 ± 14.4 μM. The IC_50_ of 5637 treated with osthole for 24 h and 48 h were 146.4 ± 8.2 μM and 68.1 ± 4.4 μM. The IC_50_ of 253 J treated with osthole for 24 h and 48 h were 160.8 ± 9.7 μM and 100.3 ± 3.3 μM (Fig. 5C). Additionally, The IC_50_ of SV-HUC-1, 5637, 253 J treated with cisplatin were also shown in Table 3. The similar phenomenon was also apparent in the colony formation of bladder cancer cells (Fig. 5D). We clearly found that the number of cell colonies was decreased as the concentration of the drug increased (Fig. 5D). In conclusion, osthole had higher toxic effects on bladder cancer cells, in addition to exhibiting lower toxic effects on normal urothelial cells.

**Table 3:** IC_50_ for bladder cancer cell line in MTT assay

**Cell line**	IC_50_(μM) for Osthole	IC_50_(μM) for Cisplatin
5637	146.4 ± 8.2 (24 h)	13.2 ± 0.7 (24 h)
	68.1 ± 4.4 (48 h)	8.14 ± 0.5 (48 h)
253 J	160.8 ± 9.7 (24 h)	17.0 ± 0.9 (24 h)
	100.3 ± 3.3 (48 h)	9.7 ± 0.6 (48 h)
SV-HUC-1	271.1 ± 13.1 (24 h)	28.2 ± 2.0 (24 h)
	211.2 ± 14.4 (48 h)	21.6 ± 1.3 (48 h)

#### Osthole promotes apoptosis in bladder cancer cells and blocks cell cycle

**Fig. 6:**
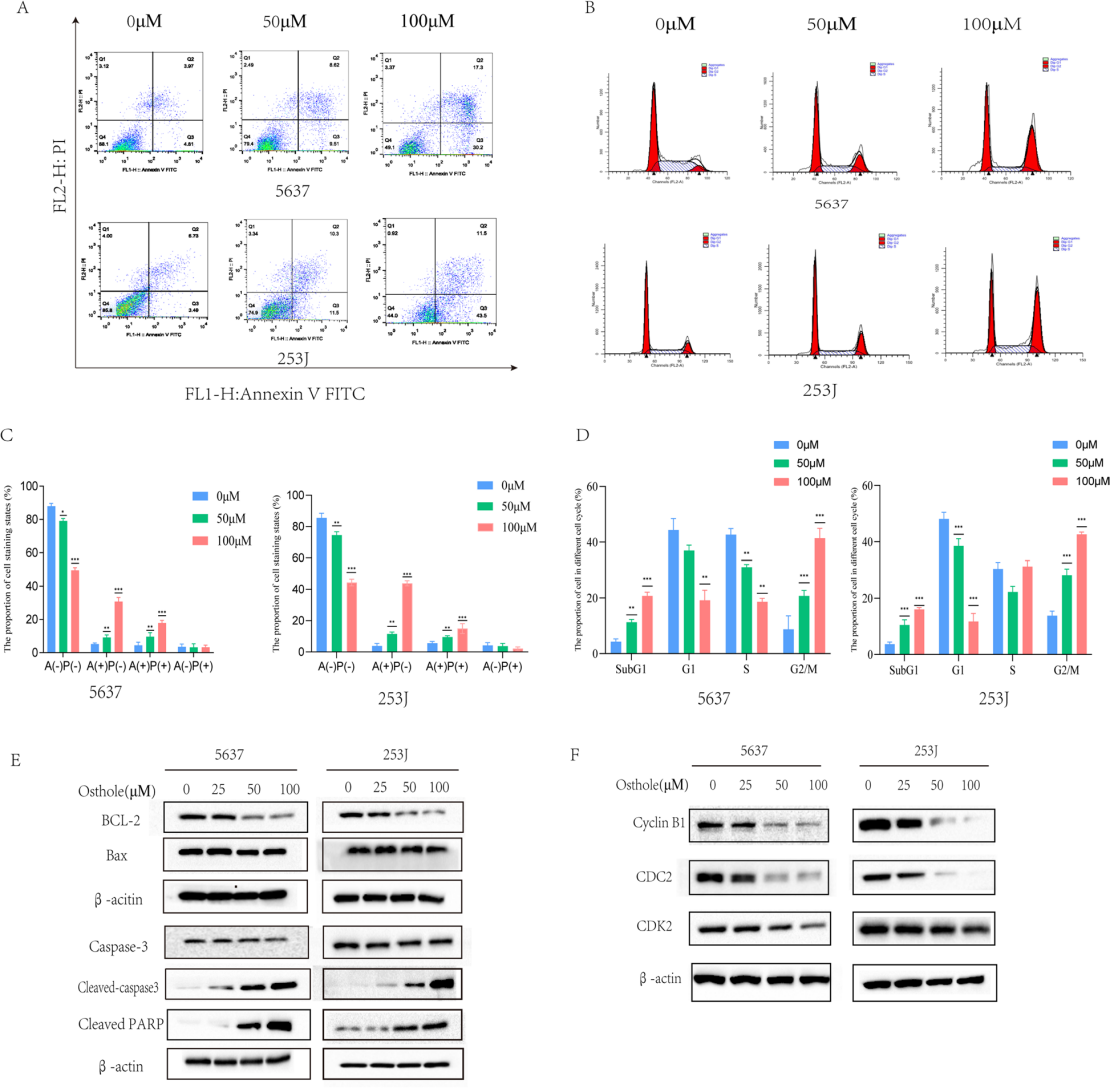
Osthole promotes apoptosis of bladder cancer cell and block cell cycle. **A** The apoptosis of bladder cancer detected by cell flow cytometry assays. **B** Bladder cancer cell cycle was detected by cell flow cytometry assays after treatment with Osthole. **C** The proportion of four cell staining states {AV(-) PI(-), AV( +) PI(-), AV( +) PI( +), AV(-),PI( +)} in bladder cancer cell. **D** The distribution of bladder cancer cell in different cell cycle. **E** The apoptosis related proteins (Bcl-2, Bax, Caspase3, Cleaved Caspase3, Cleaved parp) level were detected by western blot, β-actin was regard as control. **F** The G2 cell cycle related proteins (CyclinB1, CDC2) was detected by western blot. *p < 0.05, **p < 0.01, ***p < 0.001 compared to the control group (0 μM)

We analyzed the apoptosis and change of cell cycle in bladder cancer cells using cell flow cytometry assays (Fig. 6A, B). According to the staining method of Annexin V- FITC (AV) and Propidium Iodide (PI),we calculated the proportion of cell staining states which contained:AV(-) PI(-), AV( +) PI(-), AV( +) PI( +), AV(-)PI( +). In different four cell states, we defined the cell with AV( +) PI(-) as early apoptotic cells. Cells with AV( +) PI( +) was defined as late apoptotic cells. According the results of cytometry assays, we could clearly observed that the proportion of early apoptosis and late apoptosis in bladder cancer cells increased as the concentration of osthole increased (Fig. 6C). Simultaneously, we used western blot to detect the level of apoptosis-related proteins including Bcl-2, Caspase-3, Bax, Cleaved PARP and Cleaved caspase3. The level of Bcl-2 was gradually decreased and the level of Bax, Cleaved caspase3, Cleaved parp were gradually increased with increasing concentration of osthole (Fig. 6E). Furthermore, we also observed that osthole could block the bladder cancer cell cycle in G2/M phase (Fig. 6D). The proportion of bladder cancer cells in G2 phase gradually increased with increasing osthole concentrations. The levels of G2 phase-related proteins, CDC2 and CyclinB1, also gradually decreased with increasing osthole concentrations (Fig. 6F).

#### Osthole inhibits bladder cancer migration and epithelial-mesenchymal transition

**Fig. 7:**
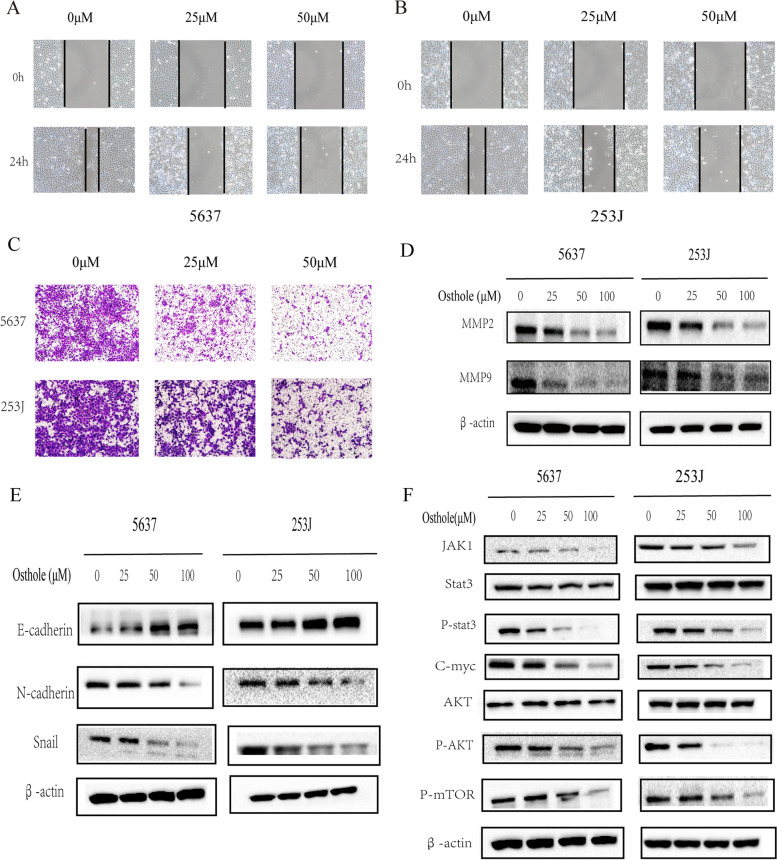
Osthole affects bladder cancer cell migration and EMT by inhibiting PI3K-AKT-MTOR and JAK-STAT3 pathway. **A** The images of wound healing assays in 5637 cell line. **B** The images of wound healing assays in 253 J cell line. **C** The images of transwell asssays in bladder cancer cells. **D** The migration and invasion related proteins (MMP2,MMP9) were detected by western blot. **E** The EMT related biomarkers (E-cadherin, N-cadherin, Snail) were detected by western blot. **F** The PI3K-AKT-MTOR pathway and JAK-STAT3 pathway related protein were detected by western blot

In order to explore the effect of osthole on the migration ability of bladder cancer cells, the concentration of osthole was set to 0, 25, and 50 μM, such that the 20% inhibitory concentration (IC_20_) did not significantly affect the proliferation of bladder cancer cells. A wound healing assay demonstrated that the osthole inhibited the lateral migration capacity of bladder cancer cells (Fig. 7A, B). Furthermore, the transwell migration assay proved that osthole inhibited the vertical migration capacity of bladder cancer cells (Fig. 7C). Osthole also decreased the levels of MMP2 and MMP9 proteins, which are involved in the bladder cancer cells invasion and migration abilities (Fig. 7D). Epithelial–mesenchymal transition (EMT) plays a critical role in bladder cancer metastasis. In addition, the protein expression of EMT markers, including E-cadherin, N-cadherin, and Snail, was influenced by treatment with differing concentrations of osthole. We could observed that the level of N-cadherin, Snail was decreased and the level of E-cadherin was increased with increasing concentration of osthole (Fig. 7E). Therefore we concluded that osthole inhibited the EMT process in bladder cancer cells.

#### Osthole blocks the PI3K-AKT-mTOR and JAK-STAT3 pathways in bladder cancer cells

**Fig. 8:**
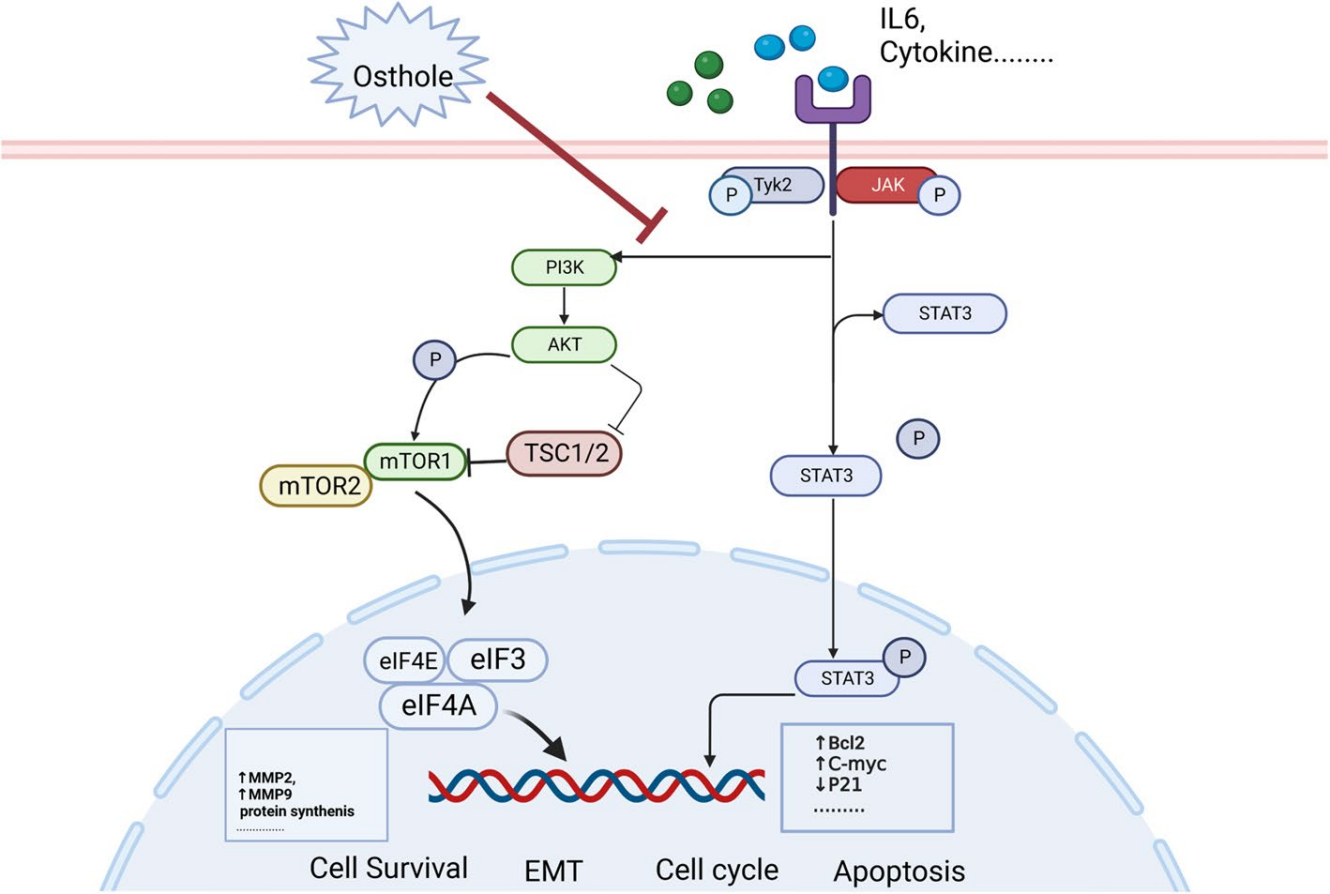
A schematic diagram of Osthole inhibits the proliferation, migration and induces apoptosis of bladder cancer by suppressing PI3K-AKT-MTOR and JAK-STAT3 pathway

The results of the KEGG pathway enrichment analysis, PPI network, and Western blot assay were used to validate that the effect osthole may have on the PI3K-AKT-mTOR pathway. Furthermore, the levels of PI3K-AKT-mTOR-related proteins, including AKT, p-AKT, and p-mTOR, were detected by western blot after treatment with different concentrations of Osthole. The protein level of p-mtor and p-akt were decreased (Fig. 7F). Meanwhile, the levels of JAK-STAT3 pathway-related proteins (JAK1, P-STAT3, and STAT3) were also decreased following treatment with Osthole (Fig. 7F). In brief, we concluded that osthole had cytotoxic effect on bladder cancer cells and inhibited migration, and EMT by blocking the PI3K-AKT-mTOR and JAK-STAT3 pathway (Fig. 8).

## Discussion

Bladder cancer is a common urinary tract cancer. Non-muscle invasive and muscle invasive bladder cancer are two distinguishable types of pathology in bladder cancer [30]. Bladder cancer recurrence following surgical resection, particularly in muscle invasive bladder cancer is high [31]. Surgery and chemotherapy have been the mainstay for bladder cancer treatment [32]. However, the chemotherapeutic drugs have high toxicity, leading to a multitude of side effects in patients, including, but not limited to anemia, liver and kidney dysfunction, and alopecia [33]. Given the high surgical recurrence rates and extreme adverse effects of chemotherapy, it is imperative to find alternative treatment options for bladder cancer. Osthole is a classical coumarin compound, which was first extracted and separated from Cnidium monnieri and other related plants [34]. Osthole is reported to inhibit various cancers. For instance, Hyocheol et al. revealed that osthole inhibits ovarian cancer by interacting with an endoplasmic reticulum-mitochondrial axis [35]. Xu et al. revealed that osthole suppresses gastric cancer cell proliferation [36]. Yin et al. demonstrated that osthole could inhibit proliferation, invasion, and migration of human cervical cancer [37]. Sun et al. revealed that osthole induces apoptosis in human tongue cancer [38].

Although accumulated research reported that osthole could inhibit various diseases. The potiential mechanism for osthole against bladder cancer was not clear. Traditional Chinese medicine research had low efficiency and accuracy. Therefore, we used network pharmacology method to predict the potential targets for osthole against bladder cancer. Then,we validated in in vitro experiment.

Network pharmacology is a discipline based on the theory of systems biology that analyzes the network of biological systems and selects specific signal nodes for multi-target drug molecule designs [39]. Recently, many scientists have screened out the effective components of drugs and potential targets using network pharmacology. Li et al. explored the active component and mechanism of the anti-gastric-cancer effect of Herba Sarcandrae [40]. Qiu et al. verified molecular evidence of the beneficial effects of licorice for the treatment of post-traumatic stress disorder using network pharmacology [41]. In addition, Ju et al. determined an underlying mechanism of osthole in the treatment of gastric cancer and screened out target genes for osthole against gastric cancer using network pharmacology [42]. However, these studies lacked experimental validation. Osthole research demonstrated the targets for osthole against bladder cancer. In this study, network pharmacology and molecular docking methods were used to locate targets for osthole and an in vitro experiment was performed to verify the cytotoxic effects of osthole on bladder cancer cells and the ability to inhibit the migration and EMT, which was differernt from other studies. According to the results of molecular results, we found that osthole had a good binding affinity to target proteins. Compared with its co-crystal ligands, osthole had lower binding energy, which validated our docking protocols.

The process of tumorigenesis are closely related to the disorder of cell cycle and abnormal apoptosis [43]. Anti-cancer drugs can induce the apoptosis of cancer cells and block cell cycle [44]. In our research, we found that osthole had cytotoxic effect on bladder cancer cells and promote the cell apoptosis according to the results of cytotoxic assay and cell flow cytometry assays. We firstly used three different cell lines to explore the effect of osthole. In addition, we found that osthole had lower toxic effects on normal urothelial cells compared with cancer cell lines. The level of apoptosis related proteins Bcl2, Bax, Caspase3 and Cleaved Caspase3 and G2/M related protein CyclinB1 and CDC2 were changed significantly.

The EMT process denotes epithelial cells being transformed into mesenchymal cells, which can promote the migration and invasion ability of tumor cells [45]. Lin et al. revealed that osthole inhibits EMT in hepatocellular carcinoma cells [46]. Wen et al. demonstrated that osthole could suppress the EMT-mediated metastatic ability in prostate cancer [47]. A similar phenomenon has also been reported in human brain cancer [48]. Accroding to the results of in our research we observed that the protein level of MMP2, MMP9 was decreased remarkably. Therefore we firstly demonstrated that osthole could inhibit the EMT process in human bladder cancer cells, which reduce the cells’ ability to invade and migrate, and metastasize.

It is important to explore the underlying mechanism of osthole against tumor cells. Accumulative research determined that osthole could inhibit various signal pathways in cancer cells. Liang et al. determined that osthole was able suppress the proliferation of endometrial cancer cells by inhibiting the PI3K-AKT pathway [11]. Yin et al. discovered that osthole could block the Wnt/β-catenin pathway, influencing the proliferation and invasion of human cervical carcinoma [37]. Dai et al. concluded that osthole could inhibit triple-negative breast cancer by suppressing Stat3 [49]. In our study, we discovered that osthole inhibited the PI3K-AKT-mTOR and JAK-STAT3 pathways in bladder cancer. The PI3K-AKT-mTOR pathway is known to play an important role in tumor cell proliferation, apoptosis, cell cycle regulation, and other physiological processes [50]. JAK- STAT3 is a critical pathway in tumor progression [51]. Under normal physiological conditions, The JAK- STAT3 pathway-related receptor is located on the cell membrane surface. When interleukin 6 and other cytokines combine with the receptor, the receptor dimerizes and activates the JAK protein and receptor. Meanwhile, the activated JAK1 protein activated the STAT3 protein and transmitted the signal to the nucleus. Interestingly, PI3K is also a kinase activated by JAK1 protein. Thus, activated PI3K could activate the AKT protein and send the signal to downstream. Activated AKT then inhibits TSC1/2 activity, while activating mTOR protein, which eventually affects transcription of key genes. In our study, we discovered that osthole could block the PI3K-AKT-mTOR and JAK-STAT3 pathways to affect the migration, apoptosis, and cell cycle of bladder cancer cells, this mechanism holds great potential in the use of osthole in bladder cancer treatment.

## Conclusions

We firstly used the network pharmacology to predict the potential targets for osthole against bladder cancer and perform molecular docking. In vitro experiment was used to validate that osthole had cytotoxic effect on bladder cancer cells and inhibited the migration, EMT process by inhibiting the PI3K-AKT-mTOR, JAK/STAT3 pathway.Above all, osthole may provide a new treatment idea for bladder cancer.

## Supplementary Information

**Additional file 1.**

**Additional file 2.**

**Additional file 3.**

## Data Availability

The datasets used and analysed during the current study available from the corresponding author on reasonable request.
