# Supplementary material for: Potential mechanisms of osthole against bladder cancer cells based on network pharmacology, molecular docking, and experimental validation
Source: BMC Complement Med Ther. 2023 Apr 17;23:122. doi: 10.1186/s12906-023-03938-5 (PMC10108473; doi:10.1186/s12906-023-03938-5)
Supplement: Supplementary file 1 — Additional file 1. [file 12906_2023_3938_MOESM1_ESM.pdf]

Molecular name	Structure	
The co-crystal ligand of MAPK1<br>5-(2-PHENYLPYRAZOLO[1,5-A]PYRIDIN-3-YL)-1H<br>-PYRAZOLO[3,4-C]PYRIDAZIN-3-AMINE	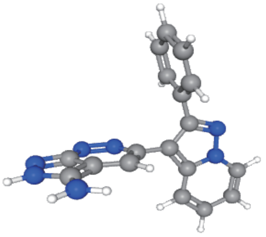	(Compound CID: 11493598)
The co-crystal ligand of AKT1<br>INOSITOL-(1,3,4,5)-TETRAKISPHOSPHATE	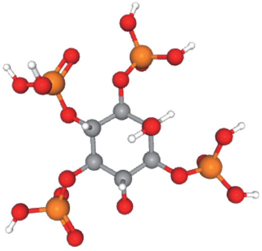	(Compound CID: 107758)
The co-crystal ligand of HRAS<br>PHOSPHOMETHYLPHOSPHONIC ACID<br>GUANYLATE ESTER	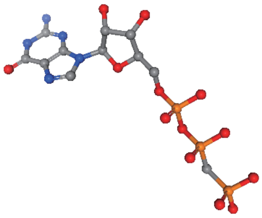	(Compound CID: 135440070)
The co-crystal ligand of HSAP90AA1<br>ADENOSINE-5'-DIPHOSPHATE	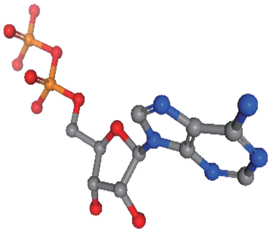	(Compound CID: 6022)
The co-crystal ligand of PIK3R1<br>O-PHOSPHOTYROSINE	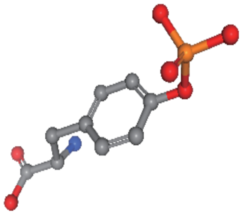	（Compound CID: 30819）
The co-crystal ligand of SRC<br>O-PHOSPHOTYROSINE	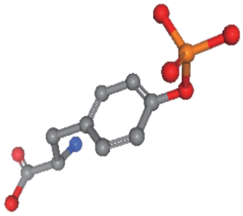	（Compound CID: 30819）

Supplementary Figure2 The 3D chemical structure of co-crystal ligand.
