# Supplementary material for: Potential mechanisms of osthole against bladder cancer cells based on network pharmacology, molecular docking, and experimental validation
Source: BMC Complement Med Ther. 2023 Apr 17;23:122. doi: 10.1186/s12906-023-03938-5 (PMC10108473; doi:10.1186/s12906-023-03938-5)
Supplement: Supplementary file 3 — Additional file 3. [file 12906_2023_3938_MOESM3_ESM.pdf]

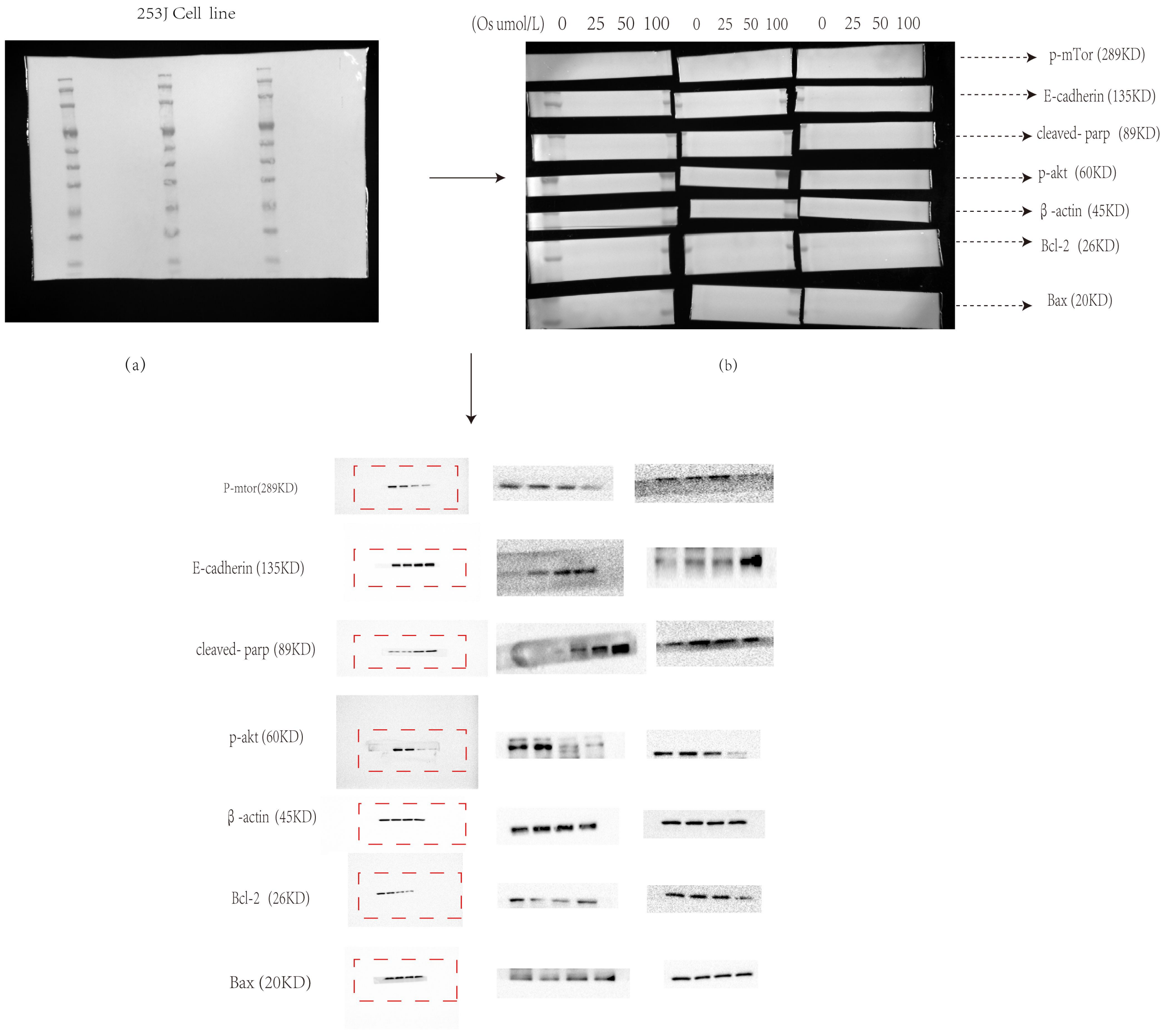


(c)

Uncropped western blots Figure1. Three independent experiments were conducted to show the results of protein of 253J cell lines treated with different concentration of Osthole (0,25,50,100umol/L), which were shown in a,b,c and cut it out from an intact PVDF membrane. The western blot of Figure6A and Figure7C,D were obtained from uncropped western blot Figure1 (b). The experiment was repeated three times.

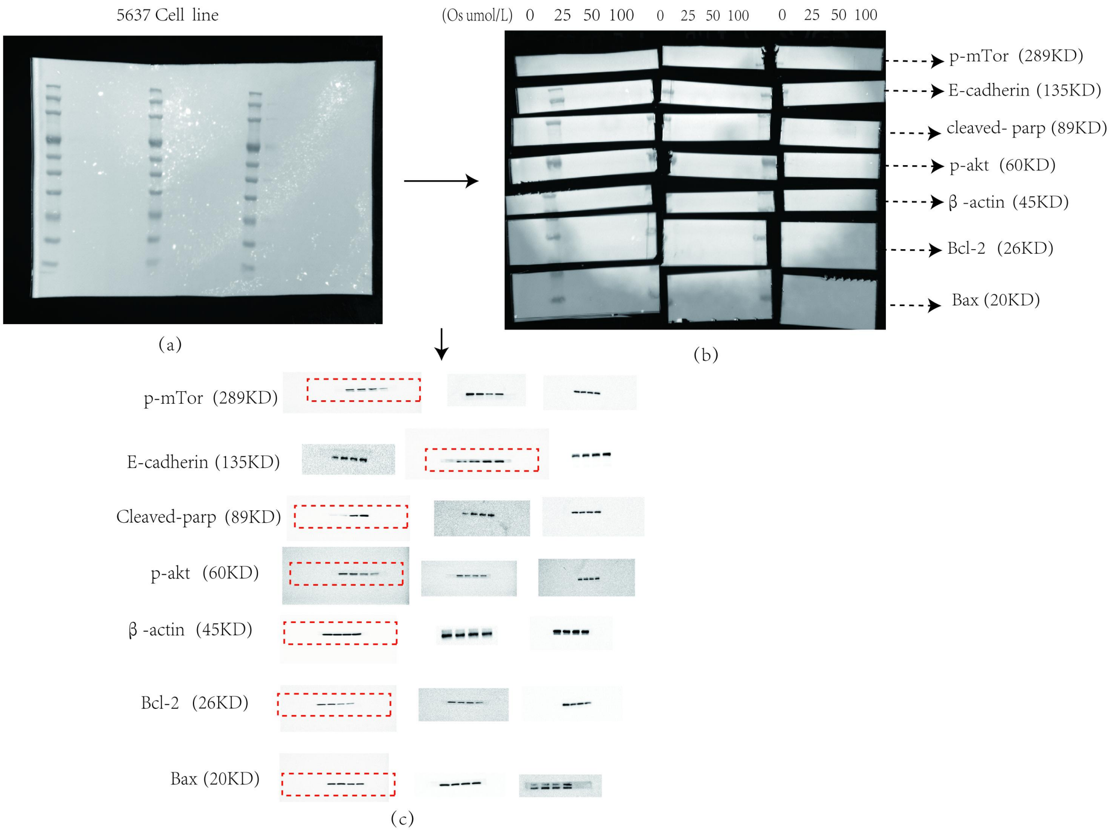


Uncropped western blots Figure2. Three independent experiments were conducted to show the results of protein of 5637 cell lines treated with different concentration of Osthole (0,25,50,100umol/L), which were shown in a,b,c and cut it out from an intact PVDF membrane. The western blots of Figure6A, Figure7C and Figure7D were obtained from uncropped western blot Figure2 (b). The experiment was repeated three times.

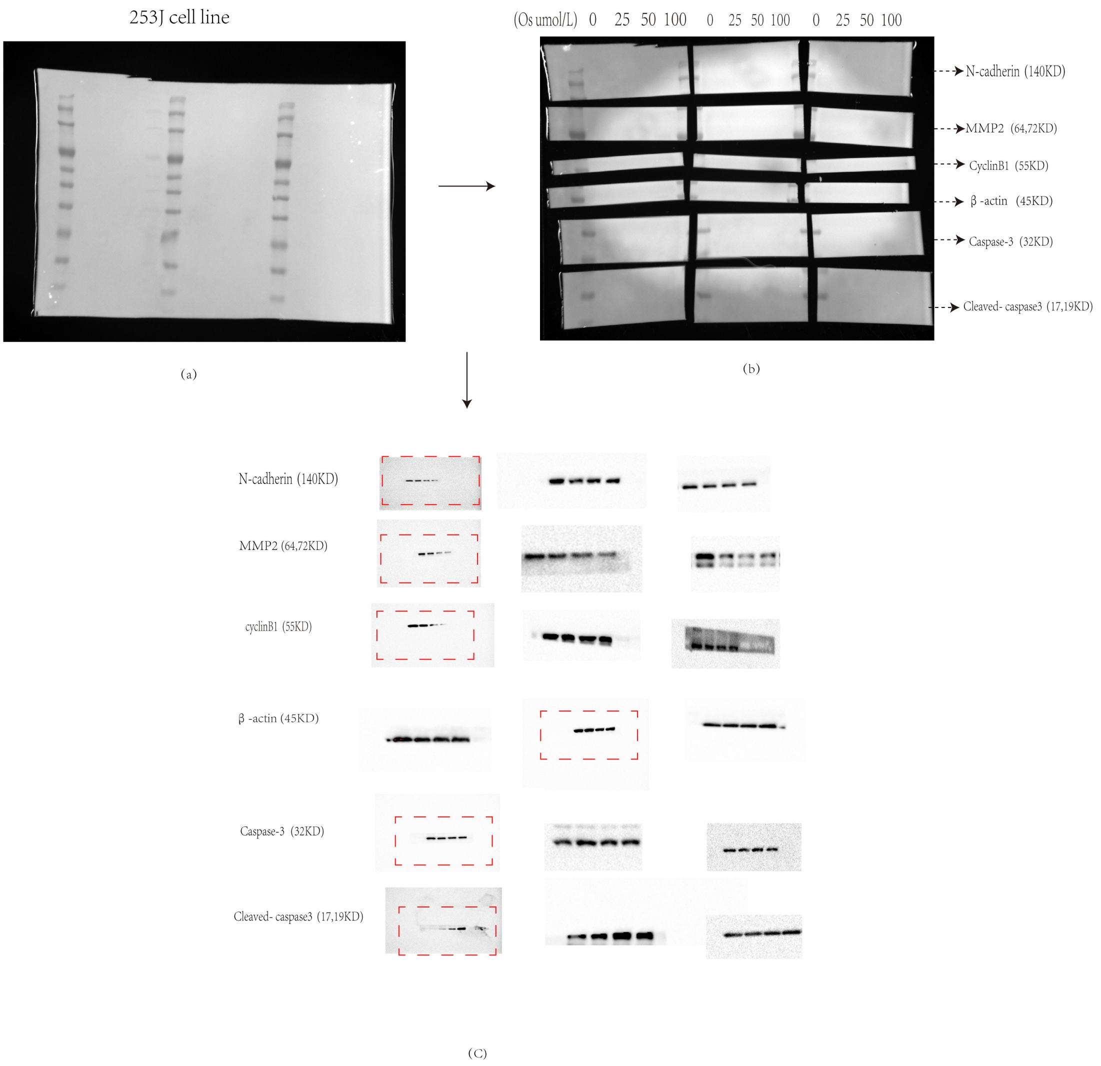


Uncropped western blots Figure3. Three independent experiments were conducted to show the results of protein of 253J cell lines treated with different concentration of Osthole (0,25,50,100umol/L), which were shown in a,b,c and cut it out from an intact PVDF membrane. The western blots of Figure6A, Figure7B, Figure7C and Figure7D were obtained from uncropped western blots Figure3 (b). The experiment was repeated three times.

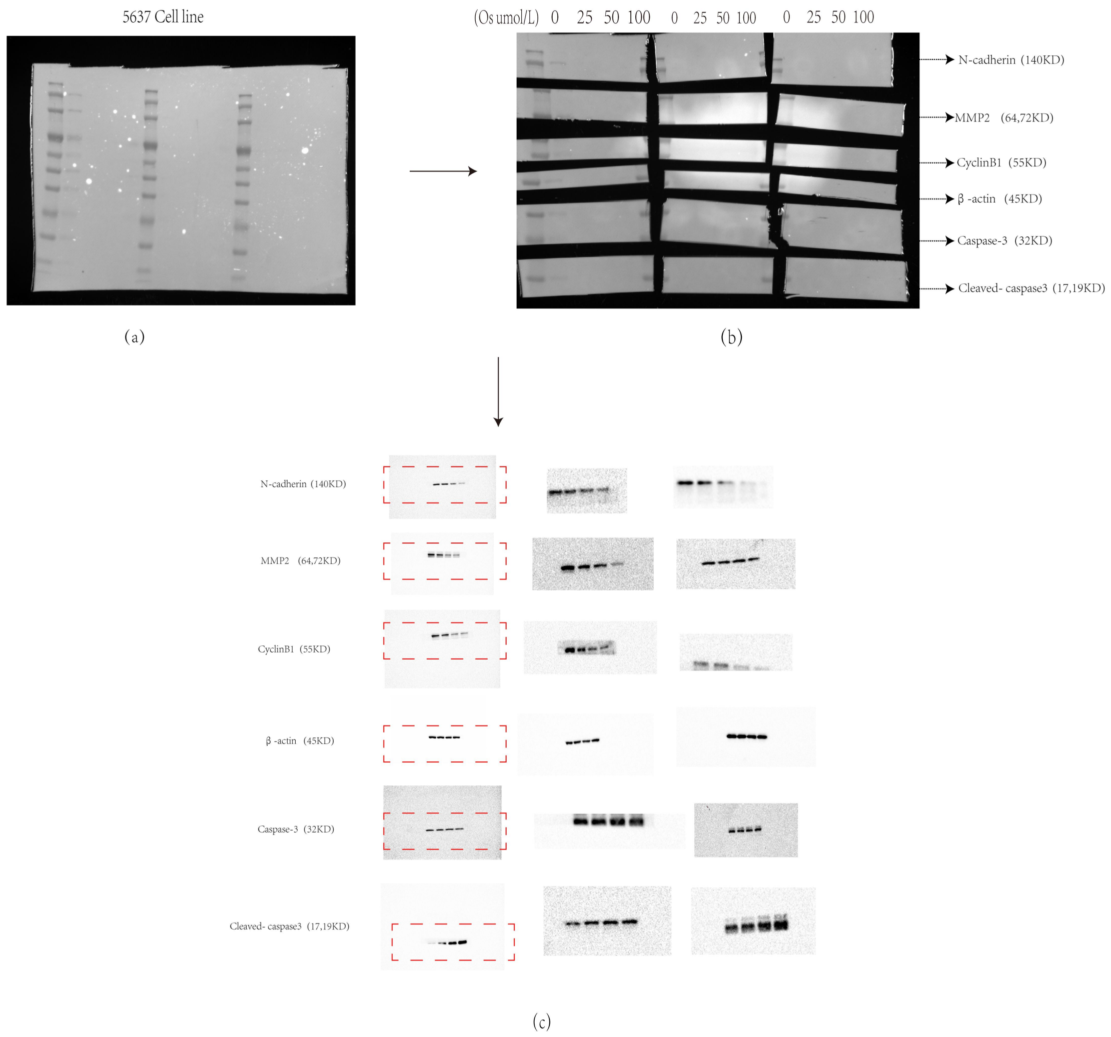


Uncropped western blots Figure4. Three independent experiments were conducted to show the results of protein of 5637 cell lines treated with different concentration of Osthole (0,25,50,100umol/L), which were shown in a,b,c and cut it out from an intact PVDF membrane. The western blots of Figure6A, Figure7B, Figure7C, and Figure7D were obtained from uncropped western blots Figure4 (b). The experiment was repeated three times.

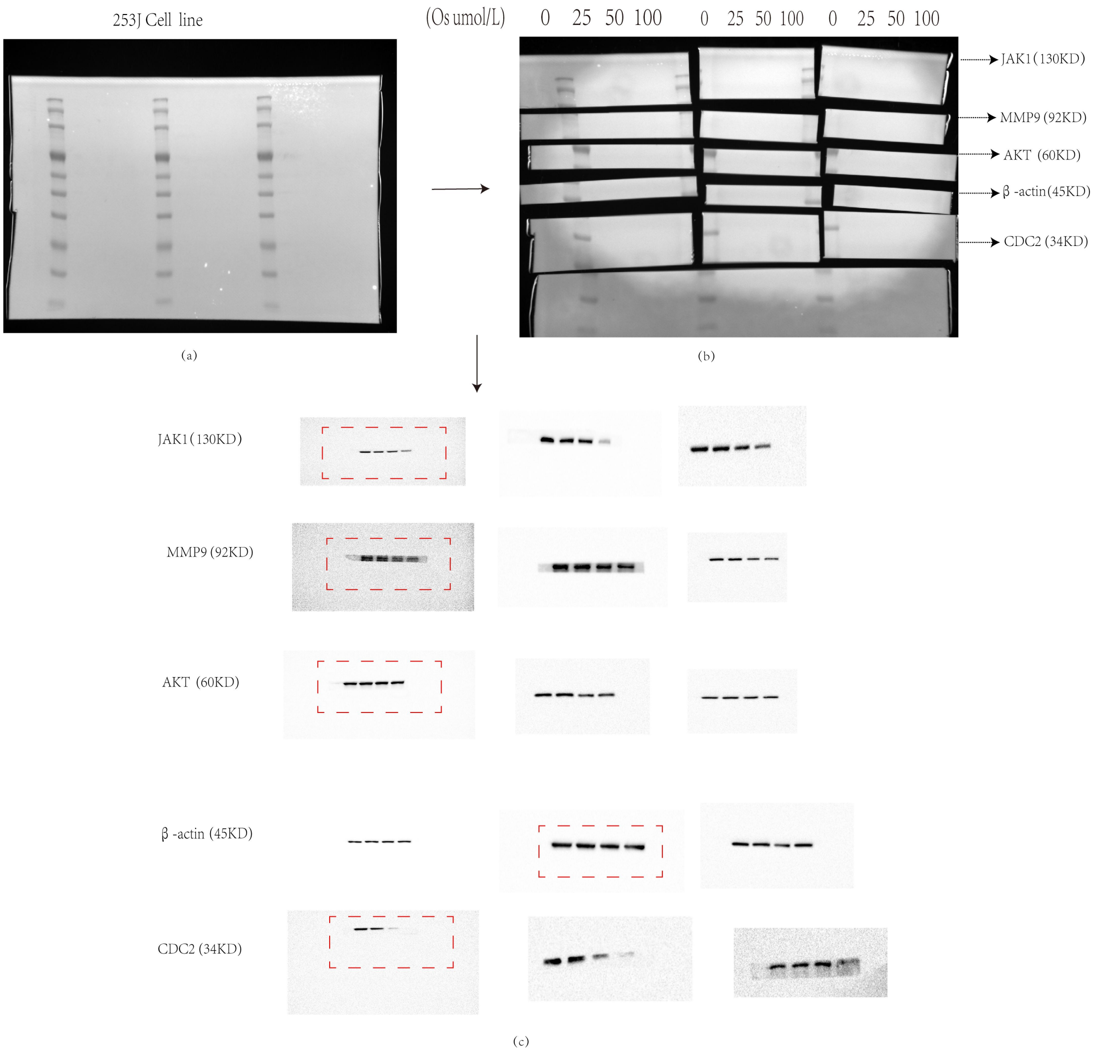


Uncropped western blots Figure5. Three independent experiments were conducted to show the results of protein of 253J cell lines treated with different concentration of Osthole (0,25,50,100umol/L), which were shown in a,b,c and cut it out from an intact PVDF membrane. The western blots of Figure6C, Figure7B and Figure7D were obtained from uncropped western blots Figure5 (b). The experiment was repeated three times.

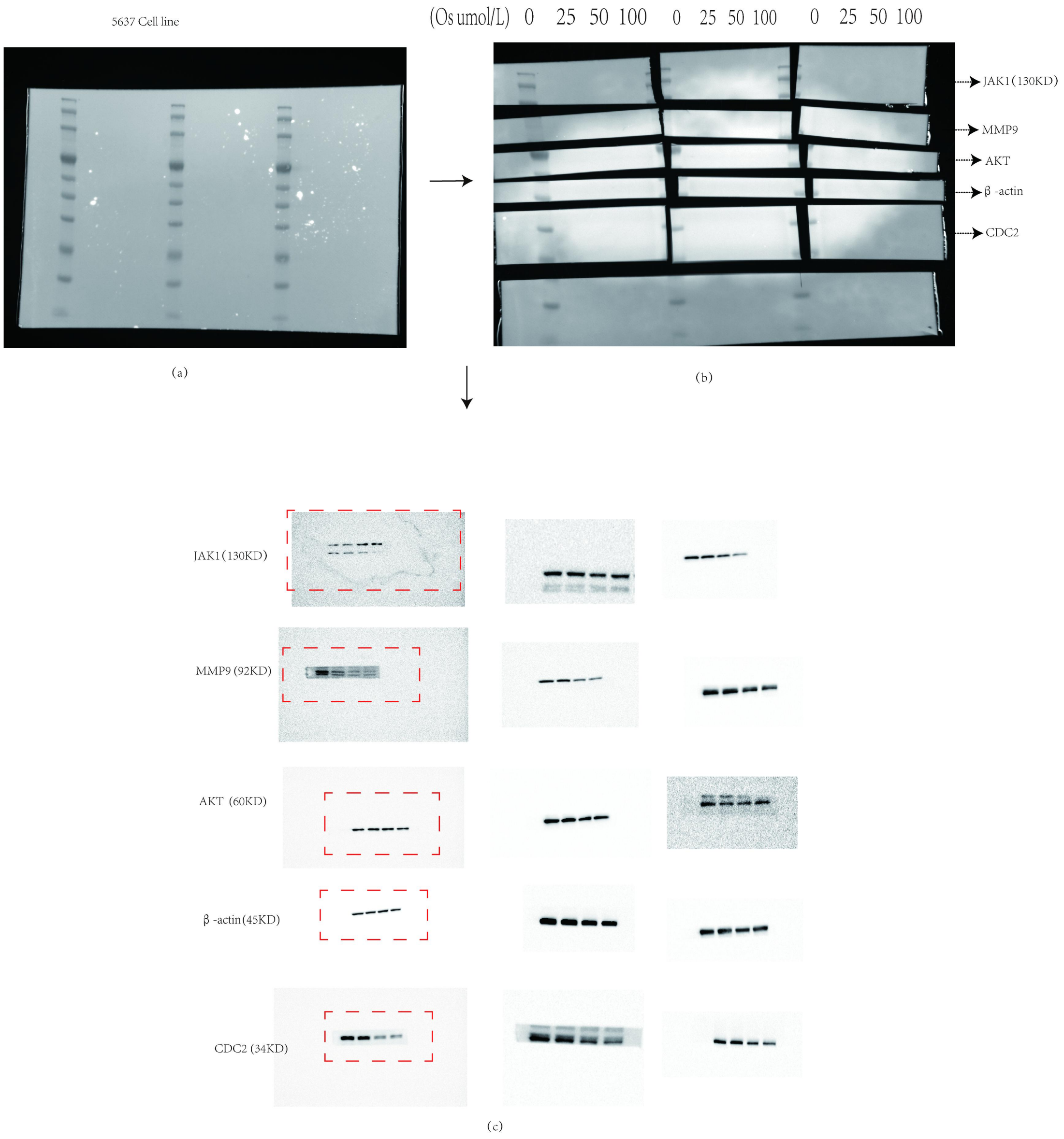


Uncropped western blots Figure6. Three independent experiments were conducted to show the results of protein of 5637 cell lines treated with different concentration of Osthole (0,25,50,100umol/L), which were shown in a,b,c and cut it out from an intact PVDF membrane. The western blots of Figure6C, Figure7B and Figure7D were obtained from uncropped western blots Figure6 (b). The experiment was repeated three times.

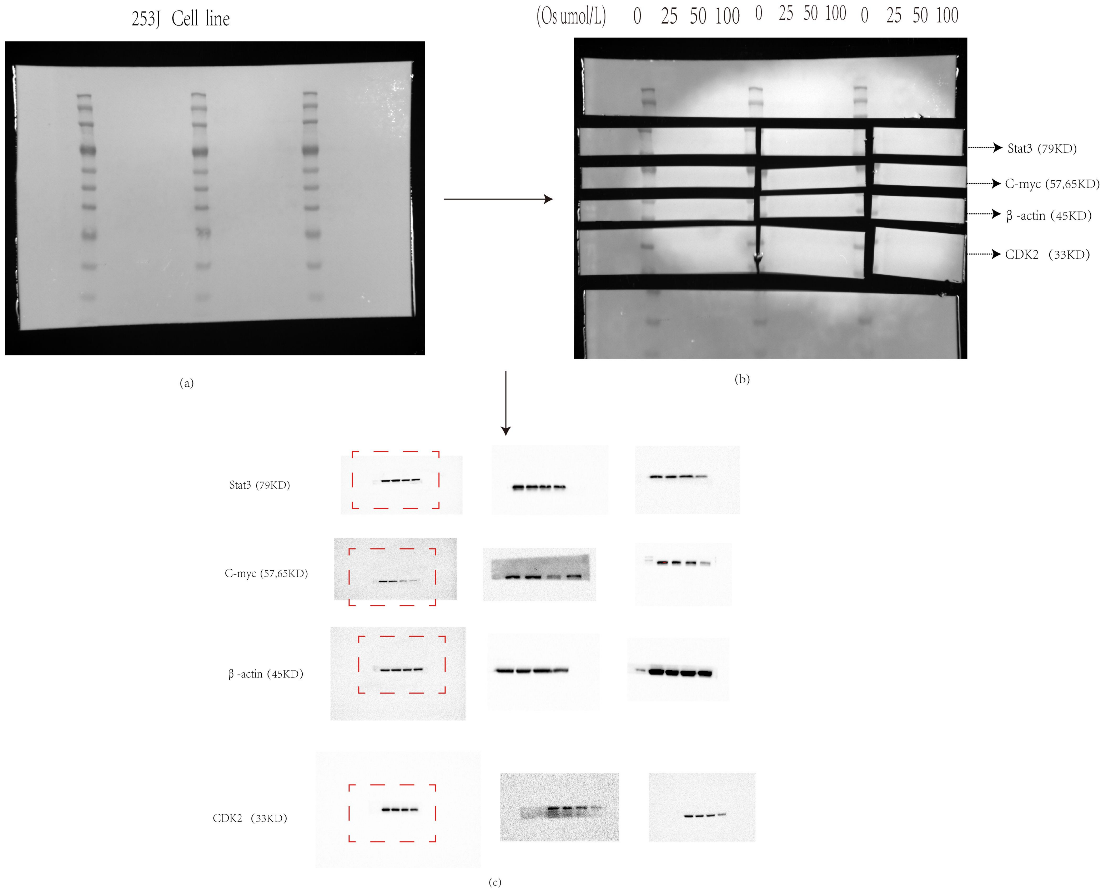


Uncropped western blots Figure7. Three independent experiments were conducted to show the results of protein of 253J cell lines treated with different concentration of Osthole (0,25,50,100umol/L), which were shown in a,b,c and cut it out from an intact PVDF membrane. The western blots of Figure6C and Figure7D were obtained from uncropped western blots Figure7 (b). The experiment was repeated three times.

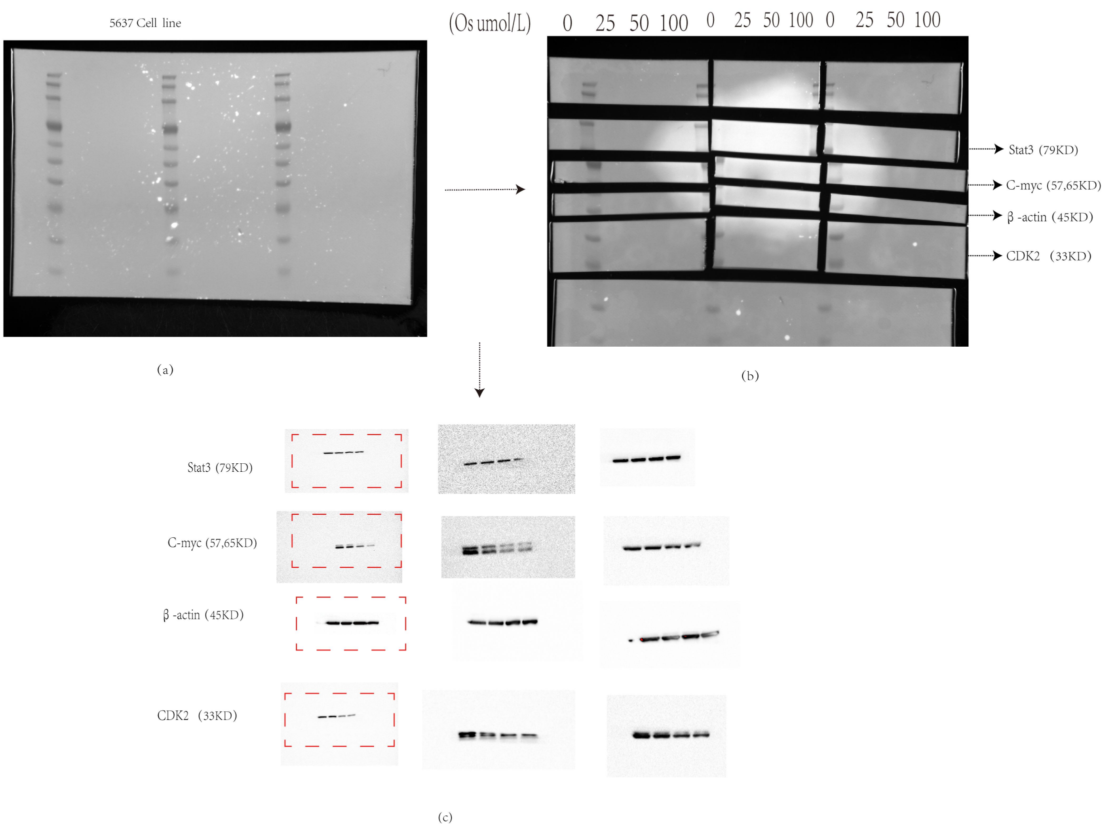


Uncropped western blots Figure8. Three independent experiments were conducted to show the results of protein of 5637 cell lines treated with different concentration of Osthole (0,25,50,100umol/L), which were shown in a,b,c and cut it out from an intact PVDF membrane. The western blots of Figure6C and Figure7D were obtained from uncropped western blots Figure8 (b). The experiment was repeated three times.

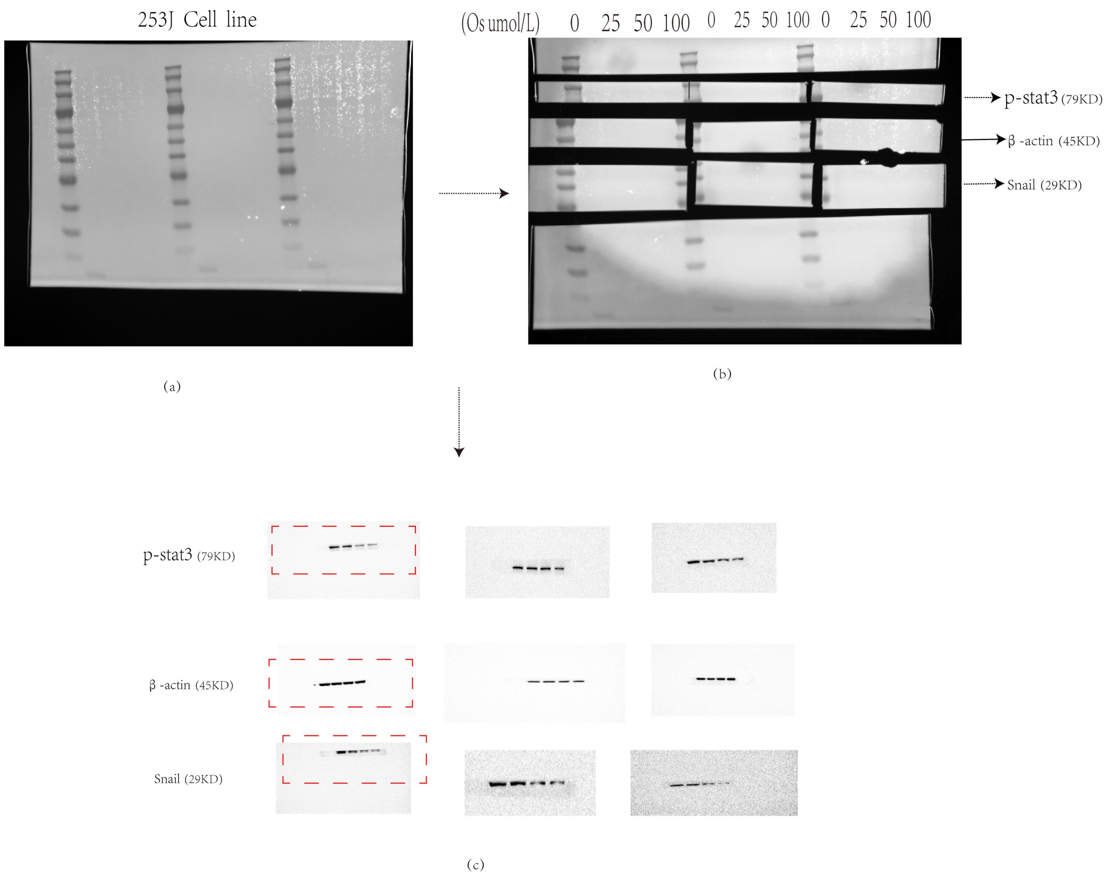


Uncropped western blots Figure9. Three independent experiments were conducted to show the results of protein of 253J cell lines treated with different concentration of Osthole (0,25,50,100umol/L), which were shown in a,b,c and cut it out from an intact PVDF membrane. The western blots of Figure7C and Figure7D were obtained from uncropped western blots Figure9 (b). The experiment was repeated three times.

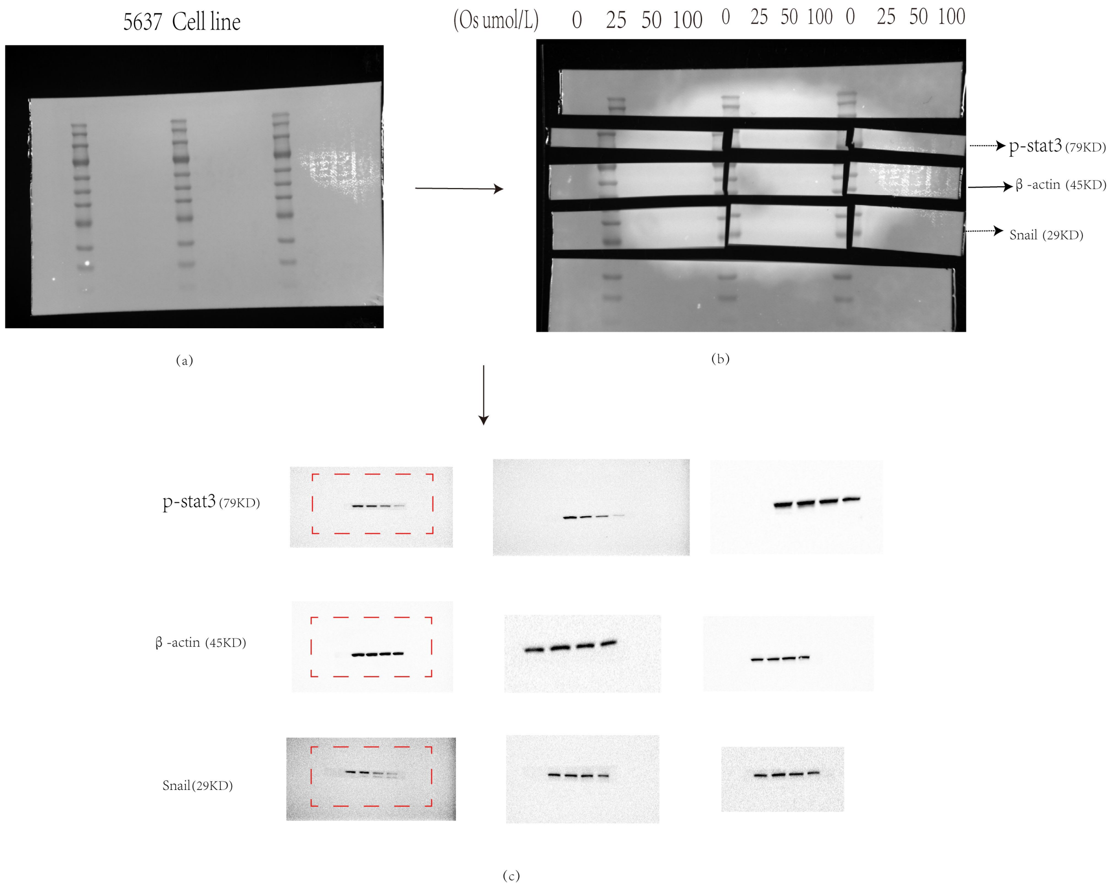


Uncropped western blots Figure10. Three independent experiments were conducted to show the results of protein of 5637 cell lines treated with different concentration of Osthole (0,25,50,100umol/L), which were shown in a,b,c and cut it out from an intact PVDF membrane. The western blots of Figure7C and Figure7D were obtained from uncropped western blots Figure 10 (b). The experiment was repeated three times.
