# Supplementary material for: Potential mechanisms of osthole against bladder cancer cells based on network pharmacology, molecular docking, and experimental validation
Source: BMC Complement Med Ther. 2023 Apr 17;23:122. doi: 10.1186/s12906-023-03938-5 (PMC10108473; doi:10.1186/s12906-023-03938-5)
Supplement: Supplementary file 2 — Additional file 2. [file 12906_2023_3938_MOESM2_ESM.pdf]

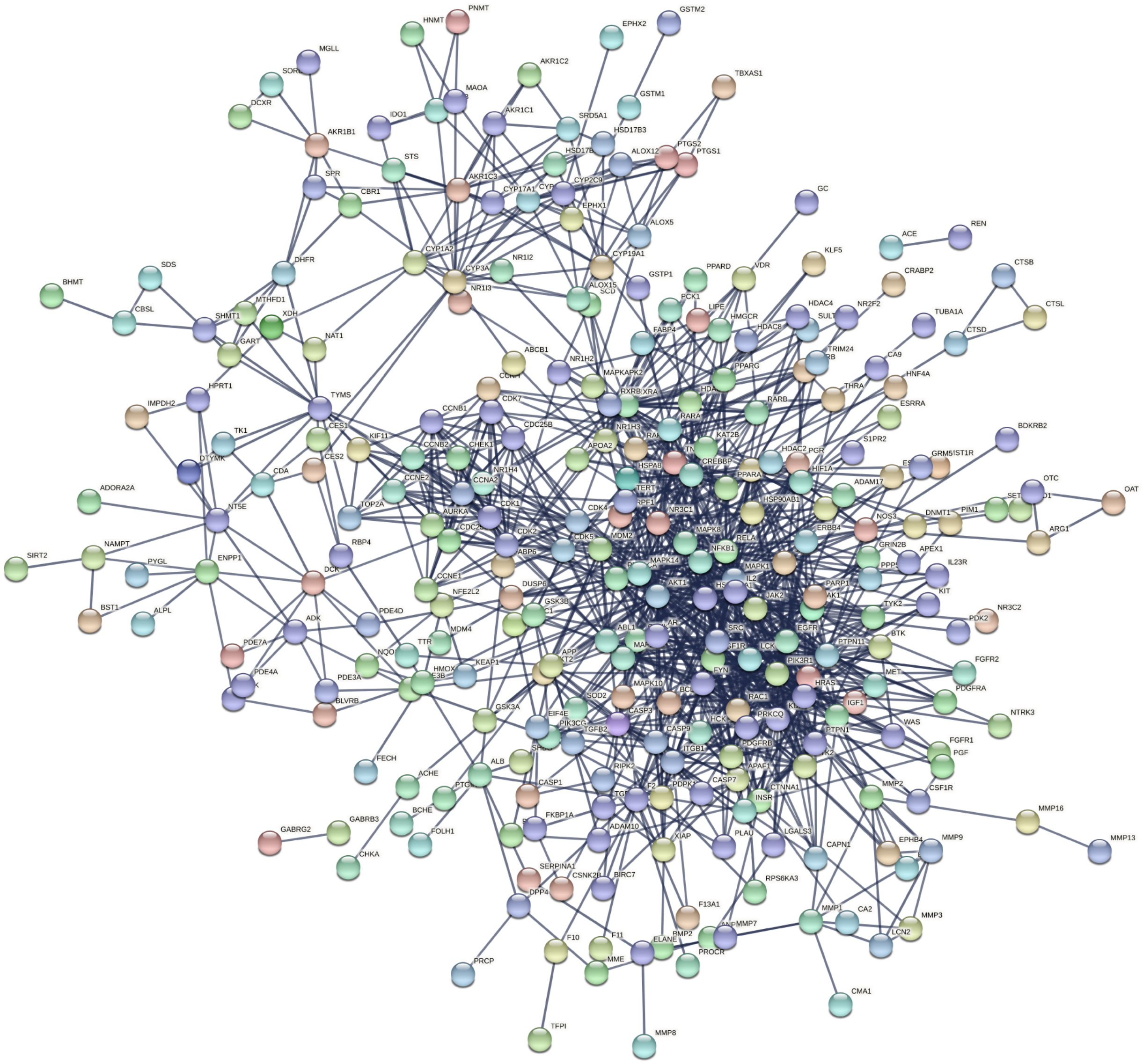

Supplementary Figure1 The PPI network of 369 target genes
